# CCAT: Combinatorial Code Analysis Tool for transcriptional regulation

**DOI:** 10.1093/nar/gkt1302

**Published:** 2013-12-22

**Authors:** Peng Jiang, Mona Singh

**Affiliations:** ^1^Department of Computer Science, Princeton University, Princeton, 08540 NJ, USA and ^2^Lewis-Sigler Institute for Integrative Genomics, Princeton University, Princeton, 08544 NJ, USA

## Abstract

Combinatorial interplay among transcription factors (TFs) is an important mechanism by which transcriptional regulatory specificity is achieved. However, despite the increasing number of TFs for which either binding specificities or genome-wide occupancy data are known, knowledge about cooperativity between TFs remains limited. To address this, we developed a computational framework for predicting genome-wide co-binding between TFs (CCAT, Combinatorial Code Analysis Tool), and applied it to *Drosophila melanogaster* to uncover cooperativity among TFs during embryo development. Using publicly available TF binding specificity data and DNaseI chromatin accessibility data, we first predicted genome-wide binding sites for 324 TFs across five stages of *D. melanogaster* embryo development. We then applied CCAT in each of these developmental stages, and identified from 19 to 58 pairs of TFs in each stage whose predicted binding sites are significantly co-localized. We found that nearby binding sites for pairs of TFs predicted to cooperate were enriched in regions bound in relevant ChIP experiments, and were more evolutionarily conserved than other pairs. Further, we found that TFs tend to be co-localized with other TFs in a dynamic manner across developmental stages. All generated data as well as source code for our front-to-end pipeline are available at http://cat.princeton.edu.

## INTRODUCTION

Transcriptional regulation controls a diverse range of biological processes, from development to response to external stimuli (1,2). Recent progress in profiling the binding landscape of transcription factors (TFs) has revealed that a single TF can bind thousands or tens of thousands of regions in a genome (3–5), and it is clear that the binding of a single TF cannot achieve the complex and precise control of gene expression exhibited in organisms (6). Combinatorial cooperativity among TFs is a central mechanism by which regulatory specificity is achieved (1,7–10). Distinct modes of cooperativity between TFs have been identified, including physical interactions between TFs for proximal co-binding (11), collaborative competition of two TFs with a nucleosome for DNA binding (12) and changes in the local conformation of DNA by one TF’s binding to assist the binding of other TFs (13,14). Further, a TF may have different sequence specificities when interacting with different cofactor TFs (15–19).

In many studies of TF cooperativity, it has been observed that certain pairs or groups of TFs tend to collaborate not only in a single region, but across many promoter or enhancer regions, following certain rules of binding motif positioning (20–24). For example, the yeast TF *MCM1* interacts with several cofactor TFs to combinatorially regulate cell cycle and mating (11,22). Its binding motif is found near those of its cofactor TFs in many regulons and in several yeast species (22). Another example comes from the Drosophila TF *dl*, which works with the TF *twist*. Binding sites for *dl* and *twist* are observed close to each other in the enhancer regions of several genes and across several Drosophila species (20,21), and binding motifs for other TFs, including *Su(H)*, also co-locate with them (24).

Given the prevalence of TF cooperativity, several computational approaches have been developed to analyze genome-scale experiments to reveal interactions between TFs. For example, ChIP experiments for TFs have been analyzed to find overlapping binding profiles (25–28) and to uncover enriched TF motifs corresponding to cofactors in distinct biological contexts (29–31) or among related species (32). Several studies have also computationally predicted TF binding sites in gene promoters from available positional weight matrices (PWM), and used these to predict combinatorial TF interactions (33–36). A common first step for all of these methods is to collect genomic binding sites for TFs. Computational methods using ChIP experiments clearly require the availability of ChIP data sets (25–27,29–32); however, ChIP data is context-specific, and given the large number of TFs in higher eukaryotes [e.g. 753 in *Drosophila melanogaster* (37) and 1700–1900 in *Homo sapiens* (38)], it is currently prohibitive to perform these experiments for all TFs in each biological context of interest. On the other hand, the large numbers of TFs in model organisms for which binding specificities are known (e.g. 364 in *D. melanogaster* and 722 in *H. sapiens*) provide a promising means for predicting binding sites for a significant fraction of TFs at the genome-scale. However, given the short lengths of binding sites for most TFs and the degeneracy in sites that a TF can bind, matches to PWMs are frequently found by chance in long genomic regions. To make higher quality predictions, binding sites are typically required to be conserved across organisms, and searched for within regions upstream of genes (39,40) or within a small set of experimentally verified enhancer regions (41,42). However, for higher eukaryotes, only a small fraction of TF binding sites are located in regions proximal to genes and a larger number of binding sites are located further away and presumably regulate transcription by higher order genome organization (43–46). For example, <20% of the *D. melanogaster* TF ChIP binding regions included in the modENCODE project overlap gene promoter regions (25). Thus, predicting binding sites only within promoter regions may miss the majority of regulatory binding sites in higher eukaryotes.

Recently, DNaseI digestion has been coupled with massively parallel sequencing to measure genome-wide chromatin accessibility and the occupancy patterns of DNA binding proteins (47–50). The binding of multiple regulators within a genomic region will increase its local chromatin accessibility to DNaseI nuclease digestion. Thus, finding DNaseI hypersensitive sites has proven to be a powerful means for mapping regulatory binding sites without requiring prior knowledge of specific DNA binding proteins. DNaseI digestion patterns have already been measured at the genome scale by high-throughput sequencing for five stages of Drosophila embryo development (48,49) as well as for 125 diverse cell and tissue types for human (50). Thus, the rapid progress of DNaseI experiments, when combined with predictions of TF binding sites, provides new opportunities for profiling genome-wide condition-specific TF occupany (51,52) as well as TF cooperativity under different conditions.

In this study, we develop a computational pipeline CCAT (Combinatorial Code Analysis Tool) to uncover combinatorially interacting motif pairs, which is designed to overcome difficulties in previous studies, including the requirement for ChIP data sets for the condition of interest or limited searching within promoter regions. We concentrate our efforts on the process of Drosophila embryo development, which involves extensive cooperativity among many TFs (1). We leverage known binding site specificities for hundreds of *D. melanogaster* TFs (3,4,39,53–60), full genome sequences for 12 Drosophila species and genome-scale chromatin accessibility data as determined by DNaseI experiments (48,49) across five conditions of embryo development. We first predict conserved binding sites for 324 TFs in these five conditions by focusing on accessible genomic regions in each condition. We show that our predictions exhibit good agreement with ChIP experiments, and are comparable in quality to high-throughput ChIP experiments, as judged via functional measures. We next search for pairs of TF regulatory motifs whose binding sites are significantly co-localized, by comparing real occurrences of binding motifs with randomized controls. We find that nearby pairs of binding sites for TFs predicted to cooperate are more evolutionarily conserved than those for TF pairs that are not predicted to cooperate, and that they tend to be found in regions bound in relevant ChIP experiments. Further, our predicted combinatorial pairs tend to be used in specific stages of embryo development, which is consistent with the dynamic nature of combinatorial regulation. The source code for our front-to-end pipeline, from predicting evolutionarily conserved genomic binding sites for TFs to uncovering preferentially co-occuring binding motifs, is available online at http://cat.princeton.edu.

## MATERIALS AND METHODS

### Searching for conserved binding sites in accessible regions

Multiple genome alignments of *D. melanogaster* and 11 other sequenced Drosophila species were downloaded from the UCSC genome browser (http://genome.ucsc.edu). Each PWM was searched on both strands of the genome sequences via the algorithm fimo from the MEME package (http://meme.nbcr.net) (61), using the default *P*-value threshold 1E-4. We excluded all binding sites in protein coding exons, as annotated by the Flybase database (http://flybase.org/).

For each match to a PWM on the *D. melanogaster* genome, we looked for matches in the other 11 genomes on either strand within an offset of 10 nt. These additional matches were considered conserved instances, and were used to calculate a branch length score (BLS) (39) as follows. We obtained the minimum phylogenetic subtree that included all conserved instances. The BLS was computed as the total branch length of this subtree as a fraction of the entire tree (39). We observed that it was possible to get a high BLS score if there was an isolated match in a species distant to *D. melanogaster*. Because such a match may be spurious, we ignored the match in the genome most distant from *D. melanogaster* if there was a gap of more than four species from the second most distant match and the evolutionary distance from *D. melanogaster* was two times bigger than the second most distant match. Then for each TF PWM, all of its binding sites were ranked by BLS scores from largest to the smallest. These BLS scores were then converted to conservation percentile scores, which represent the relative ranks among all predicted binding sites. For example, a conservation percentile score of ‘0.6’ means the current binding site has BLS score >60% of all predicted binding sites for that PWM.

For each predicted PWM binding site in *D. melanogaster*, we derived accessibility scores based on DNaseI experiments over five embryo development stages (S5, S9, S10, S11 and S14, corresponding to 3, 4, 5, 6 and 11 h after fertilization) (48,49). For each predicted PWM binding site, its DNaseI accessibility score was estimated by averaging all DNaseI experimental scores ±50 nt around it. For each stage, the top 5% of DNaseI scores across the whole genome was set as a threshold, and PWM sites with an average DNaseI score larger than this threshold were defined as accessible binding sites.

### Collection and selection of TF regulatory motifs

We collected 712 PWMs representing the binding specificities of 364 different DNA binding proteins from FlyFactorSurvey (53,54), BDTNP (4,3), Flyreg (55), JASPAR (56), Transfac 6.0 (57), a collection of Kellis and colleagues (39) and several ChIP experiment papers (58–60). For each PWM in our collection, the information content (IC) was computed for each of its columns as 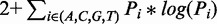, where *P_i_* gives the frequency of base *i* in the column, and columns at the beginning or the end of the PWMs with IC <0.2 were trimmed. Of the 364 collected DNA binding proteins, 170 have two or more PWMs associated with them. For example, the well-studied *bcd* has 12 different PWMs in our data set. It has been shown previously in *Saccharomyces cerevisiae* that different PWMs for the same TF may differ in quality even if they share motif similarity (62). Thus, to control for PWM quality and correct for study bias, only one PWM was selected for each TF as follows.

We collected ChIP data sets for 53 TFs from BDTNP (4,3), modEncode (63) and several publications (58–60,64,65). For any TF, if there is ChIP experimental data for it, the percent of PWM binding sites within ChIP bound regions was calculated. If several different ChIP data sets existed for the same TF, the median value was taken for comparison. For different PWMs of the same TF, the PWM with the highest ChIP percentage was selected. For TFs without available ChIP experiment, the five embryo stage DNaseI data set was utilized (48,49). In this case, for PWM selection, regions with DNaseI accessibility scores in the top 5% of scores were used in place of ChIP bound regions.

This process resulted in choosing one PWM for each of the 364 DNA binding proteins. After searching with the fimo algorithm [with the default *P*-value threshold of 1E-4 (61)], we uncovered binding sites for 324 DNA-binding proteins in the *D. melanogaster* genome.

### Clustering of highly similar TF regulatory motifs

Many TFs, especially from certain structural families, exhibit similar binding specificities (53,66). Thus predicted binding sites for different TFs may overlap extensively with each other. We clustered our selected PWMs by hierarchical clustering and then merged their overlapping binding sites.

For each pair of PWMs X and Y, their similarity was calculated using an IC weighted version of the Pearson correlation coefficient (PCC) measure as follows. For a given alignment 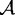 between PWMs X and Y, let 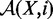 [or 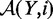] denote the column of X (or Y) corresponding to the *i*-th column in the alignment. Then, the IC of a column *i* in 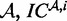, was computed as the geometric mean of the ICs, 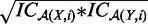, if the alignment column is ungapped or as 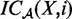 or 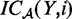 if only X or Y (respectively) contributes a column to the alignment. The weighted PCC for an alignment 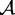 was then computed as 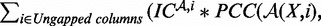 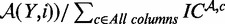, where the PCC between two PWM columns was computed over their nucleotide frequencies. In this manner, columns with higher IC contribute more to the PWM similarity score, and gaps are also penalized according to the IC of the unmatched columns. Because we search for matches to PWMs on both the positive and negative strands of the genome, the alignments between PWM X and the reverse complement motif of PWM Y were also considered. Gaps were only allowed at the ends of the alignment. A final similarity score between PWMs X and Y was calculated as the maximum possible weighted PCC over all column offsets and in both orientations.

Average linkage clustering was applied to group all PWMs into a hierarchical tree. To uncover clusters of PWMs, the tree was cut at PCC ≥ 0.8. One hundred ninety-eight PWM clusters were acquired by this threshold and 44 of them contained two or more PWMs. For each cluster of TFs, a binding site is predicted for that cluster if it is predicted for at least one of its members.

### Gene ontology enrichment analysis for the predicted TF target genes

To measure the biological function similarity between a TF and its predicted target genes, we used Gene Ontology (GO) annotations (67). We only considered GO biological process terms with >5 but <1000 genes annotated in *D. melanogaster*. For each TF and a GO term annotated with it, we computed the fraction of its annotated target genes that are also annotated with that term. For all genes that are not a target of that TF but are annotated with at least one GO term, we also calculated the fraction annotated. The enrichment ratio for that specific GO term and its annotated TF is computed as (fraction of target genes with that annotation)/(fraction of nontarget genes with that annotation). For each TF, we considered all annotated biological process terms. We use the median over all enrichment ratios across all TFs as an overall measure for each data set.

### Finding combinatorial regulatory motif pairs

Using each regulatory TF in turn as a ‘pivot’, we set out to find other regulatory motifs that significantly co-localize with it. First, we enumerated all pairs of predicted binding sites between the pivot regulatory motif and other regulatory motifs. For each enumerated pair, we defined its weight as the smaller of the conservation percentile scores of each of its constituent binding sites. For each genomic region where several binding sites for the same TF are clustered, only the neighboring sites closest to the pivot motif were considered (Supplementary Figure S1A). Then for each pivot TF motif and other motifs, we classified all enumerated binding site pairs by the distance between the two involved sites and derived a histogram in steps of 100 nt. In each histogram bin, the weights of all enumerated binding site pairs were summed as a weighted count. For this analysis, as we are using weighted counts, we considered binding site predictions with lower conservation percentile scores. In particular, we utilized those sites with conservation percentile scores ≥0.6. As shown in Supplementary Figure S6 and Supplementary Table S3, the quality of these predictions is reasonable but not as high as when using a higher threshold; however, these lower quality predictions contribute less to the weighted count.

To estimate how often TF binding sites would co-localize by chance, we randomized the identities of the other regulatory motifs. The binding site identity of the pivot regulatory motif is not changed, but the identities of all other motif binding sites are shuffled across the same chromosome (Supplementary Figure S1B). As an extra constraint in the shuffling process, we classified the 198 TF PWM clusters by the similarity in their nucleotide compositions and created 10 composition clusters (method described in a subsequent section). Only regulatory motifs in the same composition cluster can exchange their identities in the shuffling process (Supplementary Figure S2). In this way, the local base pair composition will be similar to the initial data after randomization. Then, the motif pair sites neighboring the pivot regulatory motif were enumerated again. Histograms were plotted according to the distances of all motif pairs for real data and randomized data.

Empirical *P*-values were computed in each histogram bin as the fraction of randomized weighted counts that were greater than or equal to the real weighted count among 10 000 randomizations. For each histogram bin, we only considered motif pairs with weighted count ≥1% of the total sum of all conservation percentile scores of each involved regulatory motif (pivot and other TF). The Benjamini–Hochberg procedure was applied on the empirical *P*-values, and a False Discovery Rate (FDR) threshold of 0.05 was used to select significant combinatorial motif pairs (68). The final set of combinatorial motif pairs were selected by requiring reciprocal hits when considering a distance of <100 nt (i.e. where a FDR ≤0.05 was required when each motif was used as the pivot). We also required the final set of predicted combinatorial motif pairs to have a weighted count of at least 10 where the pair sites are within 100 nt of each other and where the conservation percentile scores are ≥0.6.

### CCAT running time

We have engineered the CCAT framework so that it can uncover TF binding sites quickly. On a 2.67 GHz Intel core with 47 GB memory, CCAT takes on average 69 s to uncover conserved binding sites for fly TFs and uses <5 MB of memory. Once binding sites are determined, it takes on average 215 s to uncover other TFs whose binding sites are preferentially co-localized with it (using 10 000 randomizations) while using <50 MB of memory. We note that our framework can also be easily parallelized to run on a cluster, on a per TF basis. For example, using a Rock Cluster of 50 nodes each of the same specification as above, uncovering conserved binding sites in fly for 198 TFs and finding co-localizing TFs across the five stages took 110 min in total.

### Categorizing regulatory motifs by base pair composition

For each of the 44 PWM clusters that contained more than one PWM, one centroid PWM was generated by averaging over all included PWMs over their shared columns. Then for each of the 198 PWM clusters, we computed the A, C, G, T content of its centroid PWM by averaging over columns. The background frequency of the whole fly genome was subtracted from these compositions (A: 28.87%, C: 21.15%, G: 21.11%, T: 28.86%). The standard deviation of the entries of the resulting PWM composition frequency vector was calculated and used as a measure of base pair preference. PWM clusters whose standard deviations were in the bottom 10% when sorted by their standard deviations were excluded for further clustering, as they show only weak preferences for base pair compositions (they are found in the cluster ‘others’ in Supplementary Figure S2). All of the rest of the frequency compositions were then clustered by average link hierarchical clustering using PCC as the similarity measure. Because we search for the regulatory motifs on both strands of the fly genome, the PCCs between base pair composition vectors were calculated in both the same direction as well as the reverse complement direction, and the maximum of the two was used.

The hierarchical tree was cut at PCC 0.8. If there were small clusters with less than five members, this would lead to a restricted space in our motif identity shuffling process. In this case, we added them to the last cluster (labeled with ‘others’). Finally, 10 composition clusters were generated (Supplementary Figure S2).

## RESULTS

### Predicting TF binding sites in accessible genomic regions

We collected 712 PWMs representing 364 different *D. melanogaster* TFs from several resources (3,4,39,53–60). We searched the 12 Drosophila genomes for matches to these PWMs using the fimo algorithm from the MEME package (61). For each binding site in *D. melanogaster*, we next calculated conservation scores based on nearby matches in the 11 other Drosophila species (39,69). For each TF, only the top 20% most conserved binding sites were selected for further analysis. We next considered only conserved binding sites in *D. melanogaster* within the most accessible genomic regions, as determined by DNaseI experiments across the five embryo development stages of Drosophila (stages S5, S9, S10, S11 and S14, corresponding to 3, 4, 5, 6 and 11 h after fertilization) (48,49). For each stage, all genomic DNaseI accessibility scores were sorted from largest to smallest and the top 5% of scores were used for binding site selection.

**Figure 1.**
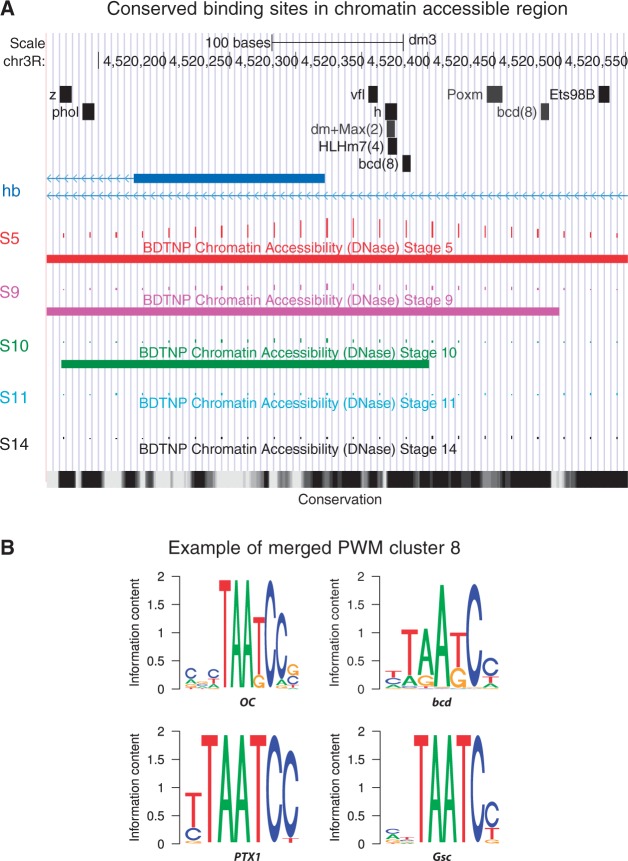
Predicted conserved TF binding sites in chromatin accessible regions. TF binding sites were predicted across the entire genome and overlapping sites were merged based on the membership of TFs within clusters of TFs with similar binding specificities. For each motif instance, the local average DNaseI accessibility scores were previously calculated for five embryo development stages (48,49). For each stage, only motif instances with DNaseI scores larger than the top 5% of all scores were selected. (**A**) Examples of motif instances near the transcription start site of the gene *hb*. Only predicted binding sites with conservation percentile scores ≥0.8 are selected. The TF names are shown over their motif instance positions. For merged binding sites, only the name of one representative TF member is shown, followed by the index number of that TF cluster. DNaseI scores for five developmental stages are shown proportional to the height of the vertical bars. Regions within the top 5% of scores are shown for each stage using horizontal bars (only in S5, S9 and S10 in this example). Conservation scores are shown on the bottom with darker colors representing higher conservation. (**B**) Example of TF cluster 8. TF members and their motif logos are shown.

We noticed that many of the collected PWMs are similar to each other [e.g. homeodomain proteins cluster into groups of TFs with similar binding specificities (53)], and thus have largely overlapping sets of predicted genomic binding sites. To address this, we grouped TFs with similar PWMs together using hierarchical clustering based on the PCC (70). This resulted in 198 TF PWM clusters, and 44 of these contained two or more TFs. Binding site predictions for any TF in one of these clusters were assumed to be putative predictions for the other TFs in the same cluster. All 44 clusters with multiple TFs were assigned indices from 0 to 43, and were referred to by that index along with a representative TF contained within the cluster. As one example, we visualized binding sites predictions near the transcription start site of *hb* (Figure 1A). While several binding site predictions correspond to a single TF, a few correspond to predictions for a cluster of TFs. For example, binding sites for TFs in a cluster including *bcd* are found; this cluster, referred to by *bcd* and the index 8, contains four different TFs including *bcd*, *oc*, *Ptx1* and *Gsc* (Figure 1B).

**Figure 2.**
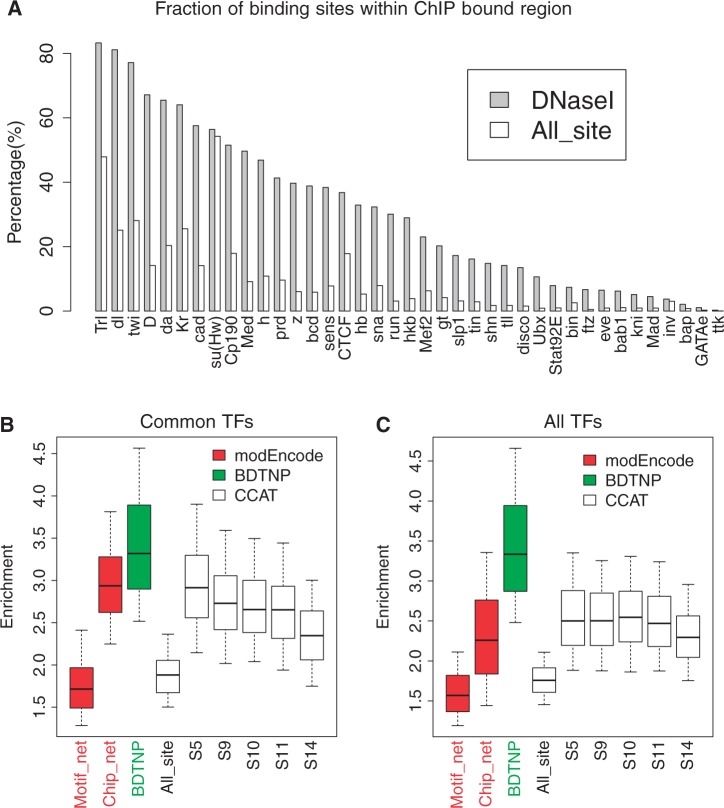
Predicted TF binding sites have quality comparable to ChIP experiments. (**A**) For each profiled TF with a corresponding ChIP data set, the percent of predicted binding sites that are located within experimentally identified bound regions was calculated. Only binding sites with conservation percentile scores ≥0.8 were considered. White bars represent the percentages calculated with TF binding sites over the whole genome. Gray bars represent percentages calculated using only binding sites within the top 5% of DNaseI scores in at least one stage. (**B** and **C**) Regulatory networks from TF to target genes were first built by connecting TFs to genes if binding sites are found within 2000 nt of the transcription start site. For each GO term (67), the enrichment ratio among target genes was calculated as the fraction of target genes annotated with the term, divided by the fraction of nontarget genes annotated with the term. For each TF, GO enrichment ratios were calculated for all of its GO biological process annotations and visualized together using boxplots for each data set. Motif_net and ChIP_net are two physical regulatory networks generated by modEncode (63). BDTNP is the regulatory network constructed from BDTNP (4). CCAT represents the networks generated by our computational predictions, constructed from conserved binding sites (conservation percentile score ≥0.8) over the entire genome or within the DNaseI accessible regions of each stage. (**B**) Only the 17 TFs profiled by all data sets are considered. (**C**) All TFs profiled in each data set are considered.

To assess the quality of our binding site predictions, we used ChIP data sets collected from diverse sources (4,3,58–60,63–65). Among 53 TFs with at least one associated ChIP data set, 39 of them are included in our TF binding site predictions. For each of these TFs, we computed the percent of its predicted binding sites that are located within ChIP bound regions. We found that by requiring conserved sites to be within a DNaseI accessible region in at least one stage, a larger fraction of binding sites are located within ChIP regions than are when considering conserved sites over all genomic regions (Figure 2A and Supplementary Figure S3).

We further compared our binding site predictions with other large-scale regulatory networks. The fly modEncode project released two physical regulatory networks: motif_net and ChIP_net. The motif_net network is computationally predicted by finding conserved binding sites within gene promoters (39,63). The ChIP_net network is determined via ChIP experiments (63). We also constructed regulatory networks from BDTNP (3,4) by assuming that a TF regulates a gene if there is a ChIP bound region within 2000 nt from the transcription start site. For our binding site predictions, we built six networks using predictions either restricted to DNA accessible regions in a specific stage or over the whole genome. Our regulatory network contains a significantly larger number of TFs (Table 1) than these previous networks.

**Table 1.** Regulatory network sizes

(A) Network level statistics				
Data set	Network	Number of regulators	Number of targets	Number of interactions
modEncode	motif_net	104	10 921	92 978
	ChIP_net	79	12 411	158 571
BDTNP	BDTNP	24	8686	43 243
CCAT	All_site	324	13 155	379 192
	S5		6503	67 153
	S9		6597	68 693
	S10		6087	62 019
	S11		6510	72 097
	S14		6631	71 305

(B) Binding site level statistics				
Network	Number of regulators	Number of targets	Number of interactions	Number of binding sites
All_site	198	13090	271 609	1 188 101
S5		6406	49 195	64 275
S9		6491	50 494	70 085
S10		5969	45 707	60 679
S11		6409	52 952	74 118
S14		6530	52 792	74 735

(A) For each data set, the number of regulators, gene targets and regulator–gene interactions are listed in each column. (B) Statistics for the data set generated by CCAT, with TFs with similar PWMs clustered and their overlapping binding sites merged.

We also assessed the quality of our network using known functional annotations from GO (67). We reasoned that the target genes of a TF should be involved in similar biological processes as it. For each GO biological process term annotating a TF, we computed an enrichment ratio by dividing the fraction of genes annotated with that term within the TF’s target genes versus the fraction of nontarget genes annotated with that term. The top 20% conserved binding sites have similar GO enrichment measures as the modEncode computational motif_net network, whereas the ChIP experimental networks ChIP_net and BDTNP have better functional quality measures than purely computationally predicted networks (Figure 2B and C). However, when restricting binding site predictions to be in the top 5% of DNaseI accessible regions, the GO enrichment measures of our binding site predictions were significantly improved in all five stages (Figure 2B and C). There were 17 TFs profiled in all four data sets. When we compared over these 17 common TFs, our binding sites predictions have similar functional enrichment ratios as the modEncode experimental ChIP network (Figure 2B and Supplementary Figure S4). When all TFs included in each network were used to compute the enrichment measures for each network, our approach obtained a higher median GO enrichment ratio than the modEncode ChIP experimental network (Figure 2C).

We also characterized the quality and size of the predicted interaction data set as the DNaseI accessibility score and the conservation percentile score thresholds were varied (Supplementary Tables S1–S3 and Supplementary Figures S5 and S6). We found that network quality increases as fewer predictions are made (Supplementary Figures S5 and S6 and Supplementary Tables S2 and S3). We chose our current thresholds (top 5% of DNaseI scores and top 20% of conservation scores) to balance the quality of our predictions with the total number of predicted binding sites (Supplementary Table S1).

As an additional quality control, we used the Redfly regulatory network, a small curated database of regulatory interactions in fly (http://redfly.ccr.buffalo.edu/). For each of the four data sets, we computed the number of interactions that overlap those annotated in Redfly and compared this against the overlap found when the Redfly network is randomized by edge swapping (71). The overlap enrichment is defined as number of overlapping interactions divided by the expected number of overlaps, as computed by averaging the number of overlapping interactions over 1000 edge-swapped (71) Redfly networks. We found our predicted regulatory network and the modEncode motif_net network consistently had higher enrichment levels than regulatory networks determined by ChIP (Supplementary Figure S7); this finding is consistent with what was reported in the modEncode project (40).

### Dynamic usage of TF binding motifs in embryo development

**Figure 3.**
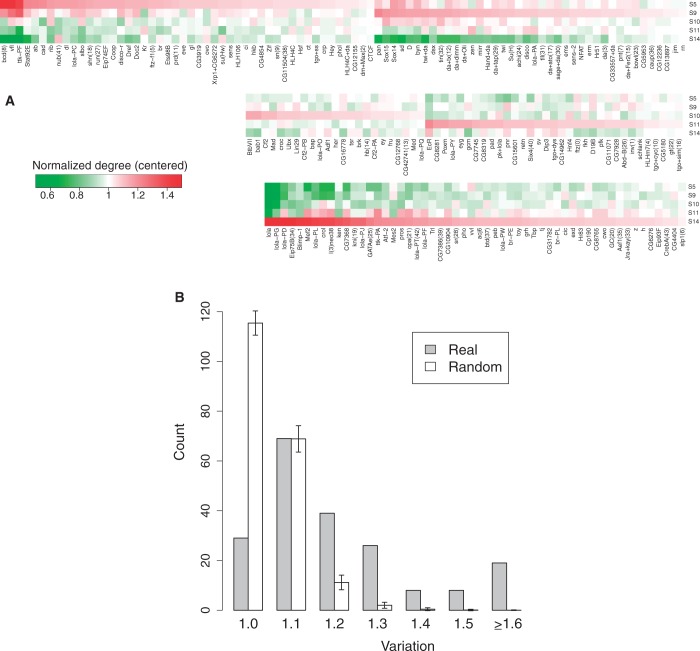
Stage-specific usage of TF binding sites across embryo development. (**A**) For each TF motif, only predicted sites with conservation percentile scores ≥0.8 were considered. The normalized degrees in different stages were calculated as the number of predicted binding sites for a given TF normalized by the total number of predicted binding sites over all TF motifs. For each regulatory motif, the centered normalized degree in each stage was computed by dividing by the average normalized degree across the five stages. These centered normalized degrees are then displayed using a heatmap. (**B**) For each TF regulatory motif, a variation ratio was calculated as the maximum normalized degree among the five stages divided by the minimum normalized degree. As a control, the DNaseI accessibility classifications for each predicted binding site were permuted across the five stages and the accessible binding sites were counted again to calculate the randomized normalized degrees and variations. Histograms for real and random variation ratios are shown, where the average and standard deviation values of random histograms were calculated from 100 randomizations.

The DNaseI accessibility data we used provides information about the dynamics of chromatin accessibility during embryo development (48,49). We used this dynamic information to determine whether binding site accessibility varies per TF across developmental progression. For each TF, we first computed its normalized degree in each stage as the number of its predicted accessible binding sites normalized by the total number of predicted accessible binding sites for all TFs (51). We found that different regulatory motifs tend to vary in their degrees across different stages (Figure 3A). For example, *bcd* has larger fractions of the accessible binding sites at the early stages S5 and S9, but lower fractions at the later stages S10, S11 and S14 (Figure 3A).

For each TF, to check the significance of its degree variation across stages, we defined the variation ratio as the maximum normalized degree across the five stages divided by the minimum normalized degree. For each motif instance, we randomly permuted the binary DNaseI accessibility determination across the five stages and counted the accessible binding sites in each stage. For each TF motif, the normalized degrees and variation measures across five stages were computed again for each randomization. We found the real data were consistently more abundant than randomized data for larger variation ratios (Figure 3B).

### Finding combinatorial regulatory motif pairs

**Figure 4.**
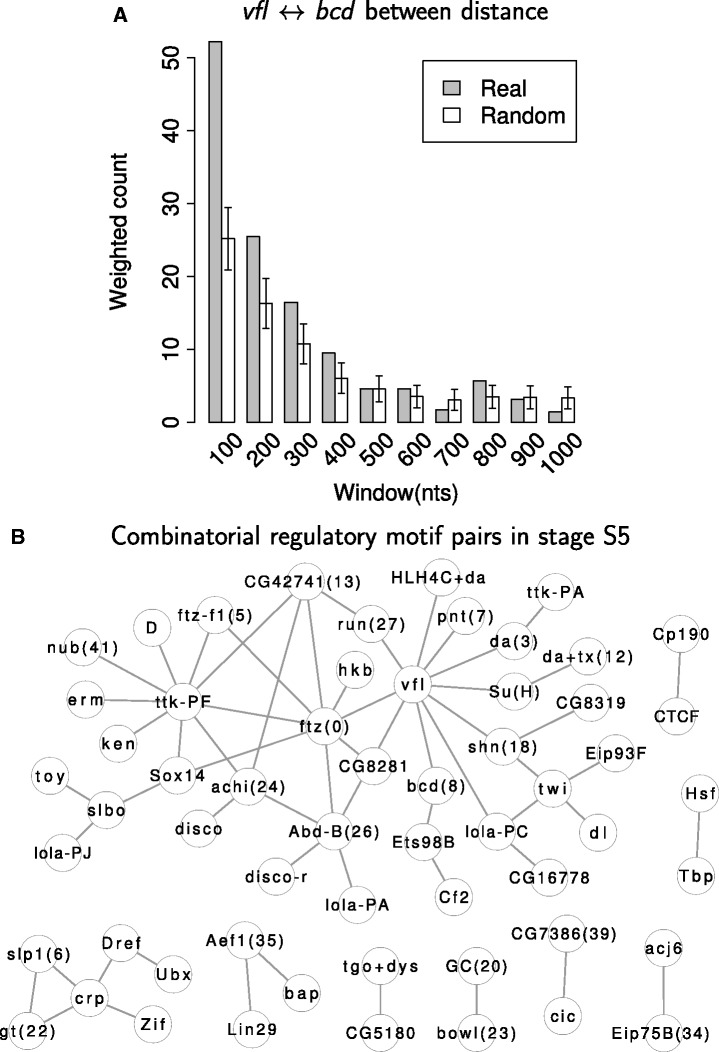
Combinatorial regulatory motif pairs with significantly co-localized sites. For each pair of TF regulatory motifs, all neighboring binding sites within DNaseI accessible regions in each stage were enumerated. The distances between all enumerated neighboring sites were profiled by a histogram with a step of 100 nt, and a weighted count in each bin was computed using the conservation percentile scores of the enumerated binding site pairs. As a background, the identities of predicted regulatory motifs with similar base pair compositions were permuted across the same chromosome and the distance histograms were profiled again. For each window, an empirical *P*-value was calculated from 10 000 randomizations, and the Benjamini–Hochberg procedure was used for multiple hypothesis correction. A FDR threshold of 0.05 was used. (**A**) For *vfl* and *bcd* in stage S5, the first window, corresponding to binding sites within 100 base pairs, has FDR <0.05. (**B**) All predicted combinatorial TF regulatory motif pairs in stage S5 are shown.

Based on our stage-specific binding site predictions, we searched for pairs of regulatory motifs that show a co-localization enrichment based on the frequency with which they occur within 100 nt of each other. For each pair of regulatory motifs, we first enumerated all predicted binding sites that fell within 1000 nt of each other (Supplementary Figure S1A). For each enumerated pair, we assigned a weight between 0 and 1 by taking the minimum conservation percentile score between the two involved binding sites. Then, we classified all enumerated pairs into distance intervals corresponding to the number of nucleotides between them (<100 nt, 100–200 nt, etc.) and summed the weights that fell into each interval (Figure 4A). To estimate the expected weighted co-localization score for each pair of regulatory motifs, we permuted the identities of binding sites among TFs with similar base pair composition (Supplementary Figures S1B and S2), while keeping the genome position and conservation percentile score associated with each site fixed. For each pair of regulatory motifs, the distribution of distances between them was computed again (Figure 4A), and an empirical *P*-value for the motif pair co-localization was computed based on the initial weighted count for motifs within 100 nt as compared with the weighted counts over 10 000 randomizations. FDRs for motif pairs were computed using the Benjamini–Hochberg procedure, and motif pairs with FDRs ≤ 0.05 were determined to be co-localizing (‘Materials and Methods’ section).

We ran the above procedure separately for each of the five studied stages and obtained 19–58 co-localizing regulatory motif pairs (Figure 4B and Supplementary Figure S8). Several previously known examples of TF cooperativity were recapitulated in this set. For example, we found several TFs that co-localize with *vfl* (also known as *zelda*), a protein critical in embryo development (72). In stage S5, we found that it co-localizes with *bcd*, which is known to be involved in early embryo development (73). Similarly, previous studies showed that *dl* and *twi* cooperate in neurogenic enhancers that direct gene expression in the early embryo (24,74), and we found that binding sites for *dl* and *twi* co-localized with each other in stage S5 (Figure 4B). In addition to capturing known cooperativity among factors that play a role in development, our pipeline also predicted co-localization between *Hsf* and *Tbp* binding sites (Figure 4B); these TFs were previously found to physically interact with each other and cooperatively bind heat shock promoters (75).

The PWMs in our collection include binding specificities for *CTCF*, *Su(Hw)* and *Cp190*, which bind insulator elements. Our CCAT pipeline found that binding sites for *CTCF* and *Cp190* co-localize in all five stages (Figure 4B and Supplementary Figure S8). Consistent with our findings, it was found that *CTCF* interacts with *Cp190*, and that its binding to targets requires *Cp190* in many cases (76). Further, the ChIP binding profiles of *CTCF* and *Cp190* were previously observed to cluster together (64).

Encouraged by the coherence of our findings with previous studies, we set out to systematically assess the quality of our predicted combinatorial motif pairs. We reasoned that if a predicted binding site for a TF is close to a predicted binding site for one of the TFs with which it cooperates, then these predictions of binding sites are more likely to be correct than other predicted sites for these TFs. To check this, we used our collected ChIP data sets. To use a TF in this assessment, for each TF that was profiled in at least one ChIP data set, we considered its top 20% most conserved genome-wide binding sites, and required that at least 5% of them be located in a ChIP bound region. If several ChIP data sets existed for the same TF, we selected the ChIP data set with the maximum percentage of predicted binding sites within ChIP bound regions.

**Figure 5.**
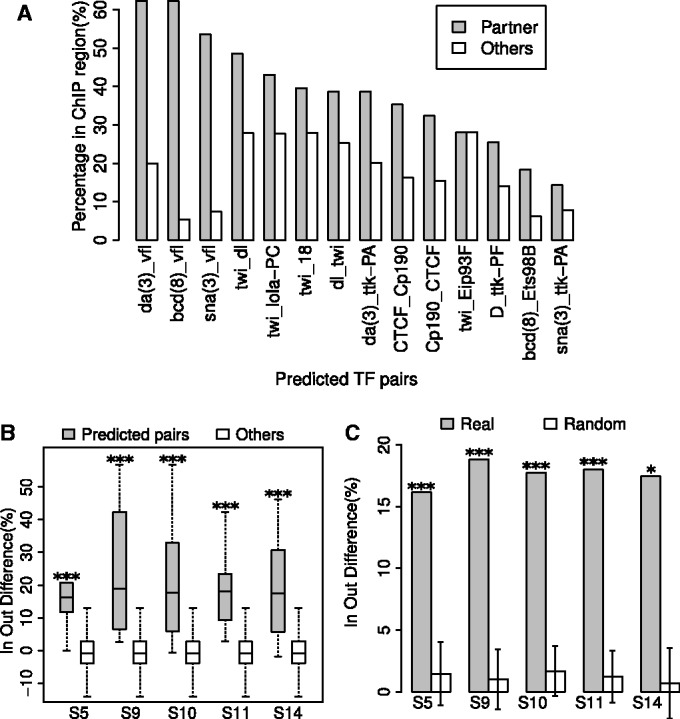
TF binding sites of combinatorial pairs are enriched in ChIP bound regions. Collected ChIP data sets are used to assess the quality of predicted motif pairs. The TF binding sites were classified into two categories: (i) those with another TF binding site within 100 nt and the neighboring TF was predicted to be a preferentially co-localized pair; (ii) those with other TF binding sites within 100 nts, but none of them comprise the predicted combinatorial pairs. For each category, the percent of TF binding sites within the ChIP bound regions was computed. (**A**) Combinatorial pairs profiled in stage S5 are considered. The ChIP percentages are plotted for the two categories. The ChIP data set that is used is specified by the first TF. (**B**) The difference of ChIP percentages between the two categories is shown for all TF pairs predicted to be preferentially co-localized and all other TF pairs that were not predicted. For each group of TF motif pairs, the measures are visualized by boxplots. The bottom and top of the box are the 25th and 75th percentiles (i.e. they give the interquartile range). Whiskers on the top and bottom represent the maximum and minimum data points within the range represented by 1.5 times the interquartile range. The Wilcoxon rank sum test was used to compare the two groups, and *P*-values were Bonferroni corrected for each of the five stages. *P ≤ 0.05 and ***P ≤ 0.001. (**C**) The combinatorial regulatory motif pairs in each stage were randomized by network edge swapping (71). For each stage, the median difference is plotted for real pairs and randomized motif pairs. Averages, standard deviations and empirical *P*-values were calculated from 10 000 randomizations. *P*-values were Bonferroni corrected for the five stages and visualized by asterisks as in (B).

For each TF considered, we classified its conserved binding sites into two categories: (i) those with a predicted conserved binding site within 100 nt of it for a TF that was found to be co-localizing and (ii) those with other predicted binding sites within 100 nt, but none of the binding sites are for TFs that were found to be combinatorial pairs. For each category, the percent of binding sites within a ChIP bound region was computed (Figure 5A).

We reasoned that if a predicted combinatorial motif pair is used at a specific stage, the first category should have more binding sites located in ChIP bound regions than the second category in that specific stage. We thus took the difference in the computed percentages for these two categories as a quality measure for the combinatorial motif pairs. For each stage, the difference in these two categories is significantly larger for predicted combinatorial pairs in all stages than for other pairs of regulatory motifs that were not predicted (Figure 5B). For each stage, we also built randomized motif pairs by treating the predicted combinatorial motif pairs as a network where edges correspond to uncovered combinatorial pairs between TFs and then randomizing the network via edge-swapping; note that this maintains the number of combinatorial pairs each motif is involved with (71). The difference measures for real motif pairs are consistently better than randomized motif pairs in all stages (Figure 5C).

We also used evolutionary conservation to assess the quality of our uncovered combinatorial pairs of TFs. A previous study in mammalian embryonic stem cells revealed that a TF binding site would be more evolutionarily conserved if it was found near a binding site for a TF with which it cooperates (77). We also searched for whether there was evolutionary constraint for our predicted combinatorial motif pairs. We first uncovered predicted binding sites for all TFs via fimo, without considering conservation. Next, for a predicted combinatorial pair involving TFs A and B, we compared the conservation percentile scores of sites partitioned into the following three groups: (i) those with motif sites of TF A and TF B within 100 nt; (ii) those with a motif site of TF A and another motif site that is not TF B within 100 nt; and (iii) those with a motif site of TF B and another motif site that is not TF A within 100 nt. Then for each of these three categories, we computed the percent of site pairs where both binding sites had conservation percentile scores ≥0.8 (Supplementary Figure S9A). Similar to the comparison based on ChIP experiments (Figure 5), we computed the difference in the fraction of highly conserved pairs between the first category and the other two categories and found that our predicted pairs consistently have more significant measures than either pairs that are not predicted or than randomized pairs (Supplementary Figure S9B and C).

### Dynamic usage of combinatorial pairs in embryo development

**Figure 6.**
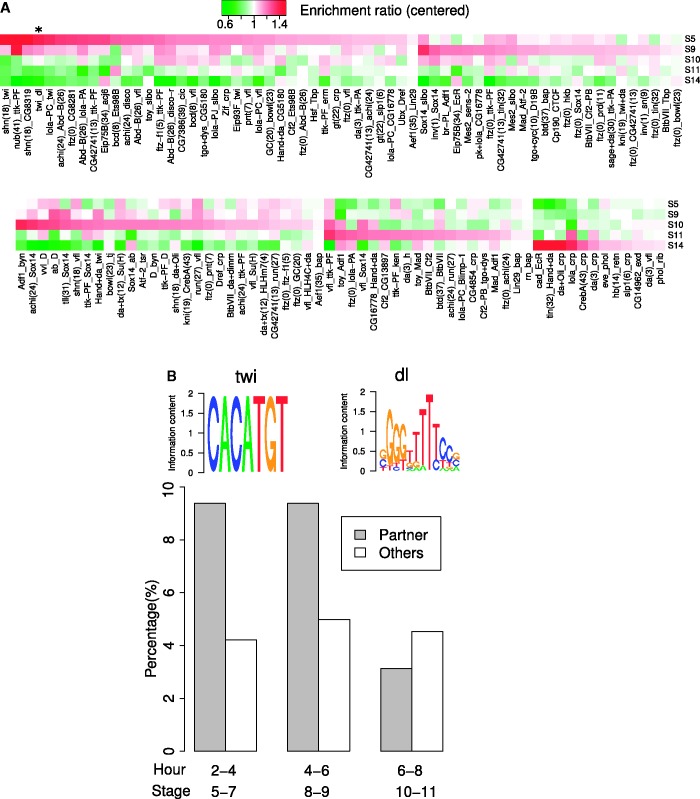
Combinatorial regulatory motif pairs are dynamically used in different stages. (**A**) For each pair of TF regulatory motifs found to be co-localized in some stage, five stage enrichment ratios were determined as (number of site pairs within 100 nt in a stage-specific DNaseI accessible region)/(number of average site pairs within 100 nt from random shuffles). For all predicted motif pairs across five stages, the enrichment ratio in each stage was centered by dividing by the average ratio across the five stages and then visualized by a heatmap. The *twi* and *dl* pair we analyze further is starred. (**B**) For the combinatorial motif pair *twi* and *dl* (highlighted by * in Figure 5A), the percent of predicted *twi* binding sites in ChIP bound regions was plotted over three consecutive stages of embryo development as in Figure 5A (58).

For all combinatorial motif pairs predicted in any of the five stages, we checked the extent to which they had stage-specific usage. We first computed the stage-specific enrichment ratio for each stage by dividing the weighted counts of site pairs within 100 nt between real and randomized data, as plotted in Figure 4A. We grouped all predicted motif pairs by their maximum stage-specific enrichment ratios and visualized them in heatmap format (Figure 6A). Certain pairs of TFs (e.g. *bcd* and *vfl*, or *twi* and *dl*) show a stage-specific preference for the earlier stages, whereas others show a preference for the later stages (e.g. *cad* and *EcR*) and some TF pairs show no apparent preference (e.g. *BtbVII* and *Tbp*).

We further concentrated on *twi* and *dl*, a combinatorial pair that we found to be strongly preferred in the stages S5 and S9 (Figure 6A), and for which we have ChIP experiments in several stages of development. In particular, *twi* has been profiled via ChIP in three consecutive embryo developmental stages (S5–7, S8–9 and S10–11) (58). We found that *twi* binding sites within 100 nt of a *dl* site had a higher fraction in ChIP bound regions in the first two stages (S5–7 and S8–9) than in the third stage (S10–11), whereas when *twi* binding sites had other TF sites nearby, the fraction within ChIP bound regions was similar across the three stages (Figure 6B). Thus, this case study is coherent with our stage-specific usage profiling (Figure 6A).

## DISCUSSION

We developed a pipeline for predicting combinatorial TF interactions based on known TF binding motifs and DNaseI data. In addition to capturing some known cases of TF cooperativity, our systematic quality assessments revealed that our predicted TF pairs are coherent with experimental ChIP data and supported by evolutionary analysis. Thus, for specific biological processes of focus, without requiring hundreds of ChIP experiments, our pipeline enabled the genome-scale profiling of the landscape of transcriptional cooperativity from a single DNaseI-seq experiment. In addition, we also developed accompanying tools to map evolutionarily conserved binding sites and to partition PWMs of different TFs into clusters of TFs with similar binding specificities, which removed redundancy in our predicted TF pairs.

### DNaseI accessibility of transcriptional repressors

It has been reported that transcriptional repressors in the human genome may not be enriched in DNaseI hypersensitive regions (50). We checked whether this phenomenon is true for Drosophila TFs by using 53 ChIP experimental data sets (Supplementary Figure S10).

For each TF with ChIP data, we computed the average stage-specific DNaseI score for each ChIP bound region and the percent of regions in each stage with average scores that are in the top 5% of genome-wide stage-specific DNaseI scores. We found that certain TFs tend to have a large fraction of their ChIP bound regions in accessible regions (i.e. in the top 5% of DNaseI scores), whereas other TFs tend to have a small fraction of their ChIP bound regions in accessible regions. As an example, the TF *sbb*, which is annotated as a transcriptional repressor by GO (67), has <2% of its bound regions accessible in each of the five embryo development stages (Supplementary Figure S10A). Of the 53 TFs with ChIP data, we determined the TFs annotated by GO as either having function ‘positive regulation of transcription, DNA-dependent’ or ‘negative regulation of transcription, DNA-dependent’, but not both, and obtained 9 and 10 TFs in each of these categories, respectively. For each of these sets, we considered the average fraction of ChIP bound regions within DNaseI accessible regions and found that the set of positive regulators tends to bind accessible regions more frequently than the set of negative regulators (Supplementary Figure S10B, *P* = 0.008 by Wilcoxon rank sum test). Thus, it may be that the strategy of using DNaseI experiments to help uncover functional TF binding sites may be better suited for activators than for repressors.

### Positional constraints between regulatory motifs

Currently, we only considered proximity in binding sites when predicting whether two TFs combinatorially cooperate. However, several studies have shown that within enhancer regions, TF binding sites may follow specific positioning and orientation rules (14,33,78). For example, in the human Interferon-β enhancer, eight TFs bind together with a specific motif order within 55 bps of DNA (14). Further, in several well-characterized developmental enhancers in Drosophila, binding sites show a periodic distribution that reflects the geometry of helical turns of DNA (78). A recent paper came out that finds positional constraints in Drosophila, but it appears to find largely homotypic interactions, which we do not consider (79). A computational study in yeast also showed that interacting TF binding sites follow strict spacing and orientation preferences (33).

To date, we have not found evidence that our predicted combinatorial motif pairs exhibit any spacing or orientation preferences between binding sites. Instead, we observe a relatively flexible spacing in our data, consistent with the billboard model of enhancers (7). For example, one study of Drosophila cardiac development revealed that five TFs cooperatively bind a large set of enhancers that have diverse motif compositions along with flexible positioning between binding sites (80).

One possibility for the reported differences in positional constraints might come from the biological processes studied. Our study is focused on embryo development and it is possible that developmental combinatorial binding allows flexible spacing. On the contrary, the Interferon-β enhancer needs to rapidly respond to viral infection (14) and may prefer a highly ordered structure among TF binding sites. Another possibility for differences in positional constraints might come from the evolutionary differences between organisms. In yeast, protein physical interactions between TFs might facilitate the strength of cooperativity, and the spacing and orientation constraints reflect the constraints of physical interactions (33). In higher eukaryotes such as Drosophila, spacing flexibility might allow TF cooperativity in more enhancers and allow more cooperativity with different TFs. Finally, it may be that more sensitive statistical approaches are able to detect support for positional constraints. However, without a systematic study of TF motif positioning under many different biological contexts, it is not possible to conclude whether flexible or strict positioning is more common in combinatorial regulatory motif pairs.

### Systematic profiling of combinatorial regulatory codes across diverse biological processes

We have shown that combining binding sites matches to TF PWMs with DNaseI accessibility experiments can result in high-quality genome-wide TF binding site predictions that are comparable in quality with those obtained by ChIP experiments (Figure 2B and C). Several previous studies also reached a similar conclusion (46,52,81). Our high-quality binding site predictions allowed us to find combinatorial interactions between regulatory motifs at the genome scale. The different conditions of DNaseI experiments also enabled us to uncover the dynamic usage of combinatorial motif pairs in different stages. Compared with ChIP experiments, which require one experiment for each TF in each condition, DNaseI experiments enable genome-scale profiling of combinatorial codes in a single experiment. The recent release of the ENCODE project includes genome-wide DNaseI experiments for 125 different human cell lines and tissue types (50). Thus, the growing availability of DNaseI experiments should enable reliable combinatorial regulatory motif pair profiling in many biological conditions.

## SUPPLEMENTARY DATA

Supplementary Data are available at NAR Online.

## FUNDING

National Science Foundation [ABI-0850063]; National Institutes of Health (NIH) [NIGMS R01 GM076275]. Funding for open access charge: NIH [NIGMS R01 GM076275].

*Conflict of interest statement*. None declared.

## Supplementary Material

Supplementary Data
