# Supplementary material for: CCAT: Combinatorial Code Analysis Tool for transcriptional regulation
Source: Nucleic Acids Res. 2013 Dec 22;42(5):2833–47. doi: 10.1093/nar/gkt1302 (PMC3950699; doi:10.1093/nar/gkt1302)
Supplement: Supplementary Data [file supp_42_5_2833__index.html]

CCAT: Combinatorial Code Analysis Tool for transcriptional regulation — CCAT: Combinatorial Code Analysis Tool for transcriptional regulation — Supplementary Data 

# CCAT: Combinatorial Code Analysis Tool for transcriptional regulation

## Supplementary Data

files

**Files in this Data Supplement:**

- Supplementary Data - pdf file
