# Supplementary material for: CCAT: Combinatorial Code Analysis Tool for transcriptional regulation
Source: Nucleic Acids Res. 2013 Dec 22;42(5):2833–47. doi: 10.1093/nar/gkt1302 (PMC3950699; doi:10.1093/nar/gkt1302)
Supplement: Supplementary Data [file supp_gkt1302_nar-02146-n-2013-File008.pdf]

Supplementary Figure S1 - Finding combinatorial regulatory motif pairs

(A) Enumerating neighbor motif pairs. In case 1, two binding sites of motif A are close to the binding site of B. Since only the closest motif pair is considered, 1 pair is counted. In case 2, the binding site of motif A is close to the binding site of motif B. However, since another binding site of A overlaps with site B, 0 pairs are counted. (B) Motif identity shuffling. Each regulatory motif was considered separately as a pivot. The identities of all other regulatory motifs were randomly permuted within each composition cluster (listed in Supplementary Figure S2), and significant proximal motifs for the pivot motif were profiled. For the final predicted list of preferentially co-localized binding site pairs, a reciprocal mutual hit was required as both TFs should identify each other as proximal.

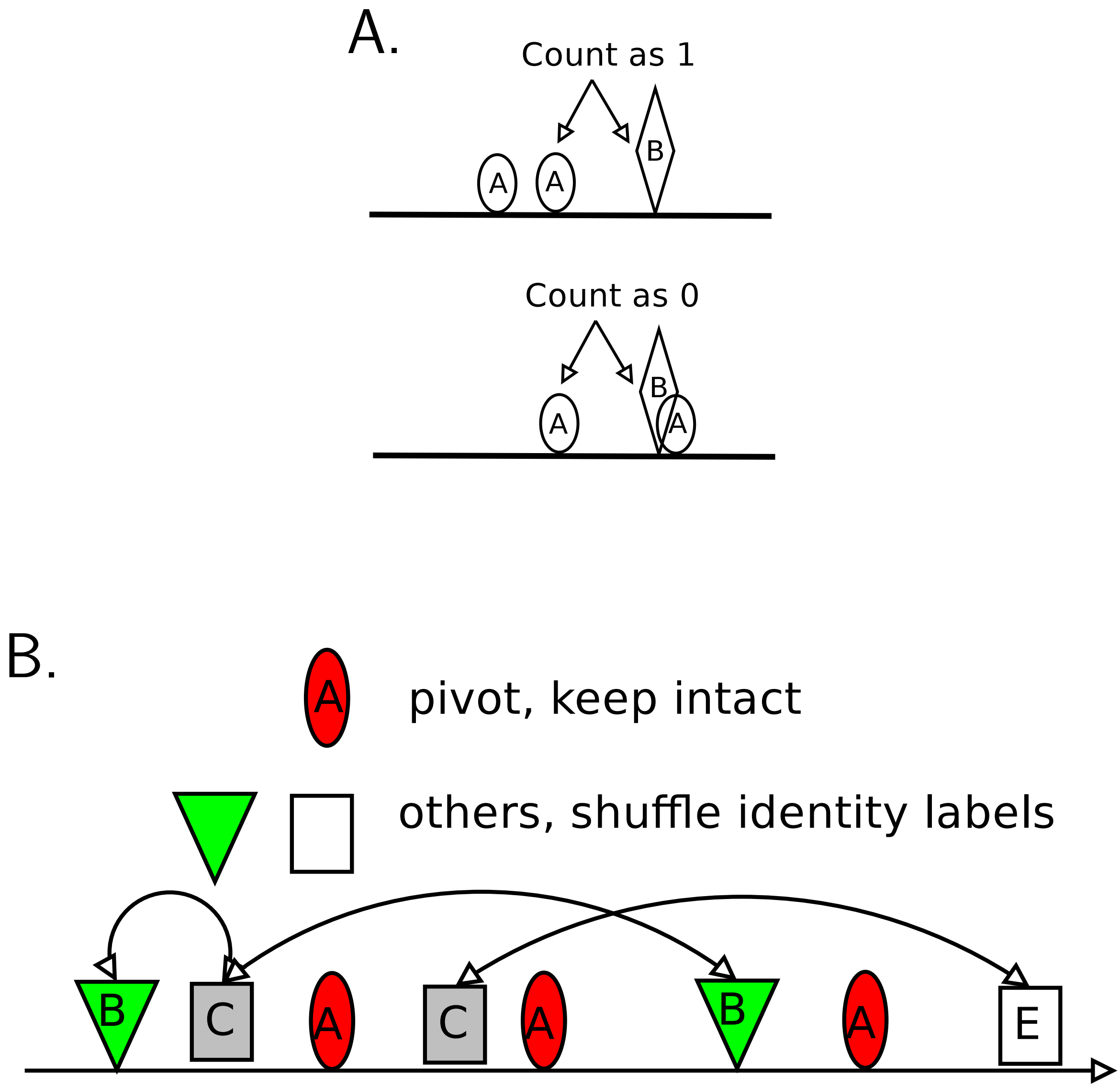

Supplementary Figure S2 - Clusters of regulatory motif base pair compositions

For all 198 TF regulatory motifs, we clustered them by the similarity of their base pair compositions. Each PWM was converted to a frequency vector of their A,C,G,T content minus the background frequency of A,C,G,T over the whole fly genome. The standard deviation of each PWM composition was computed as a measure of base pair composition bias. The bottom 10% of them were excluded from further clustering as they do not have strong preferences for compositions (shown with the cluster “others”). Then, all of the remaining composition vectors were clustered by average link hierarchical clustering based upon the Pearson correlation coefficient. The hierarchical tree was cut at a Pearson correlation of 0.8.

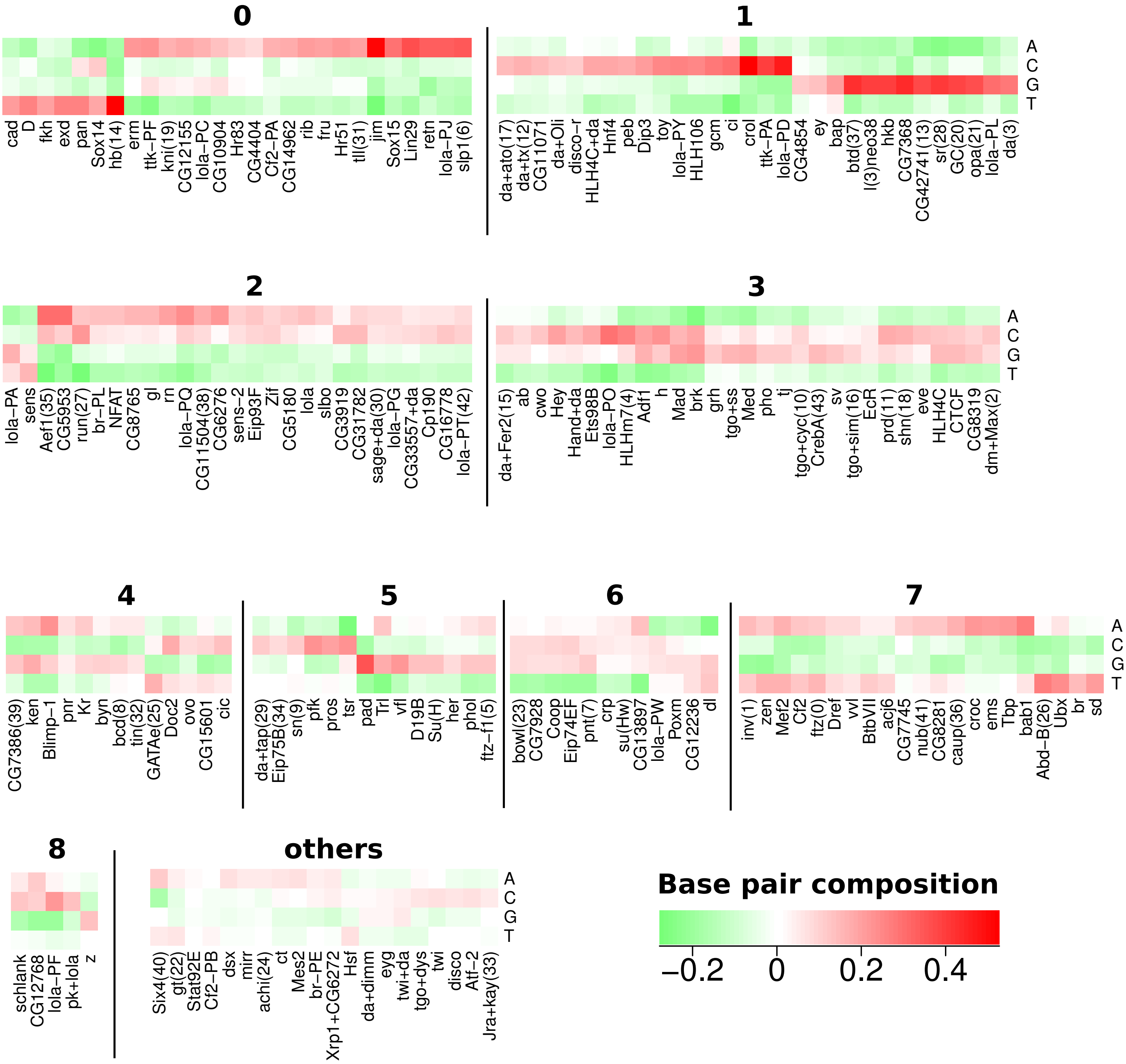

Supplementary Figure S3 - The CCAT predicted binding sites significantly overlap with ChIP experiments

We selected predicted binding sites within the top 5% of accessible DNaseI scores in at least one stage and having conservation percentile scores greater than or equal to 0.8. For each TF, we computed the fraction of these sites that fell into ChIP bound regions and compared these with data obtained via randomizations. In particular, for each predicted binding site, the TF identity was randomly swapped with another TF if both of them were profiled in our set of collected ChIP datasets. The standard deviations from 10 randomizations were represented by error bars.

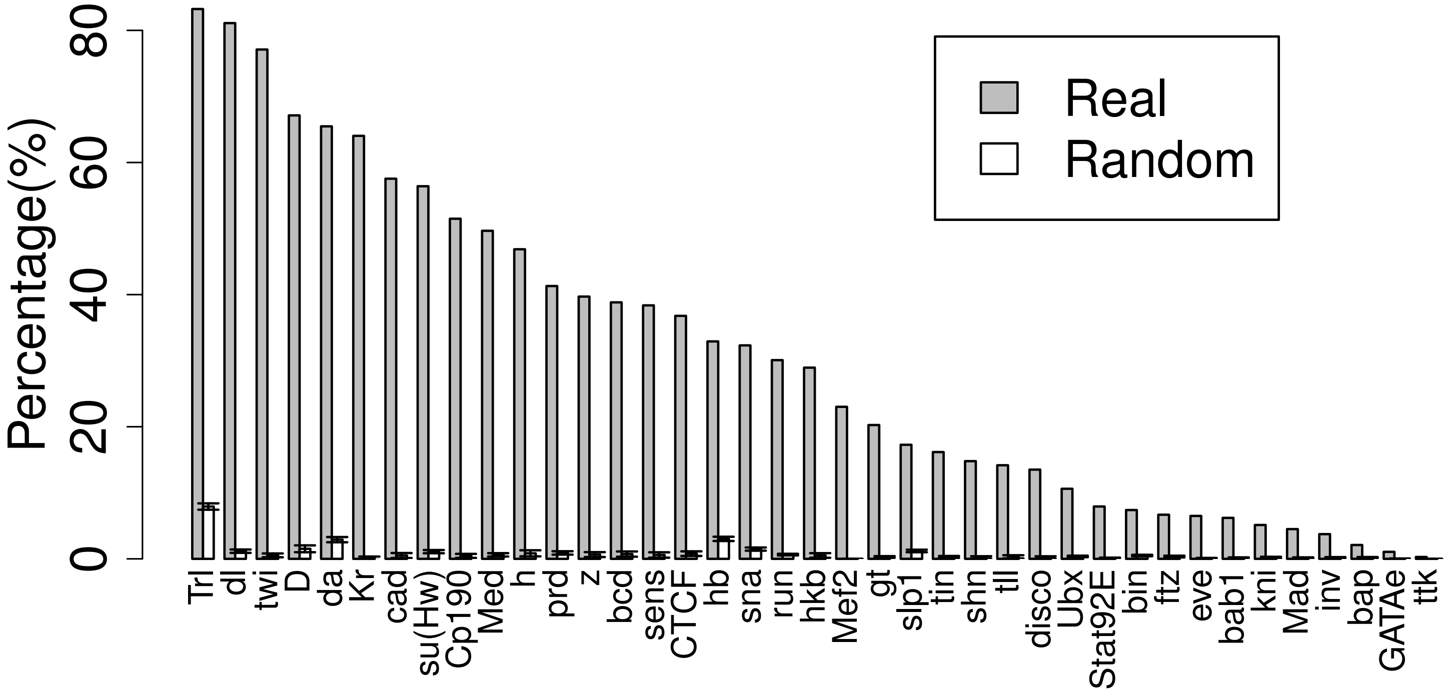

Supplementary Figure S4 - GO enrichment assessments for regulatory network targets

For each TF and its annotated GO biological process terms, GO enrichment ratios among target genes were calculated and visualized as described in Figure 2B. Only 17 TFs that are included in all datasets are shown.

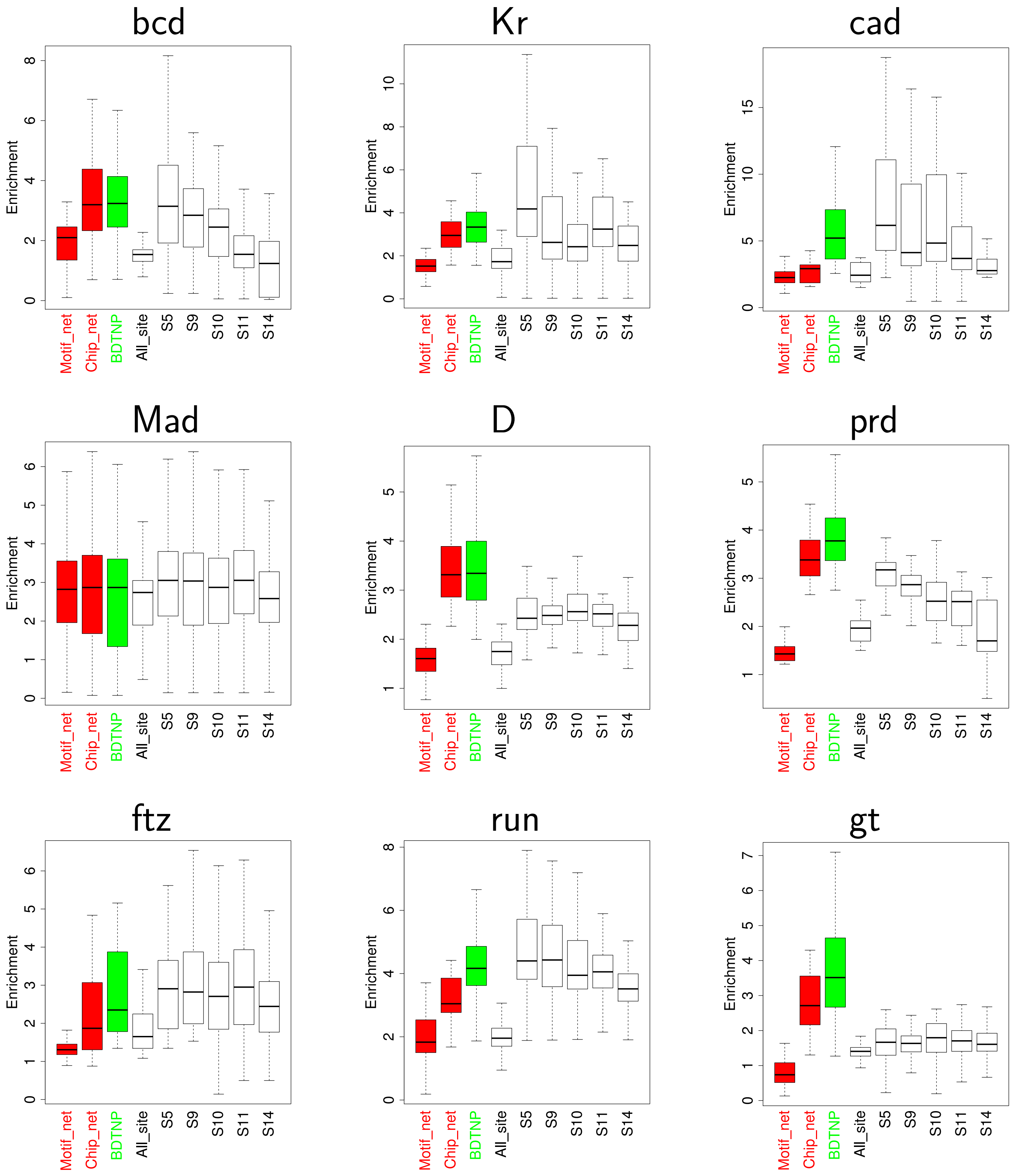

Supplementary Figure S4. continued

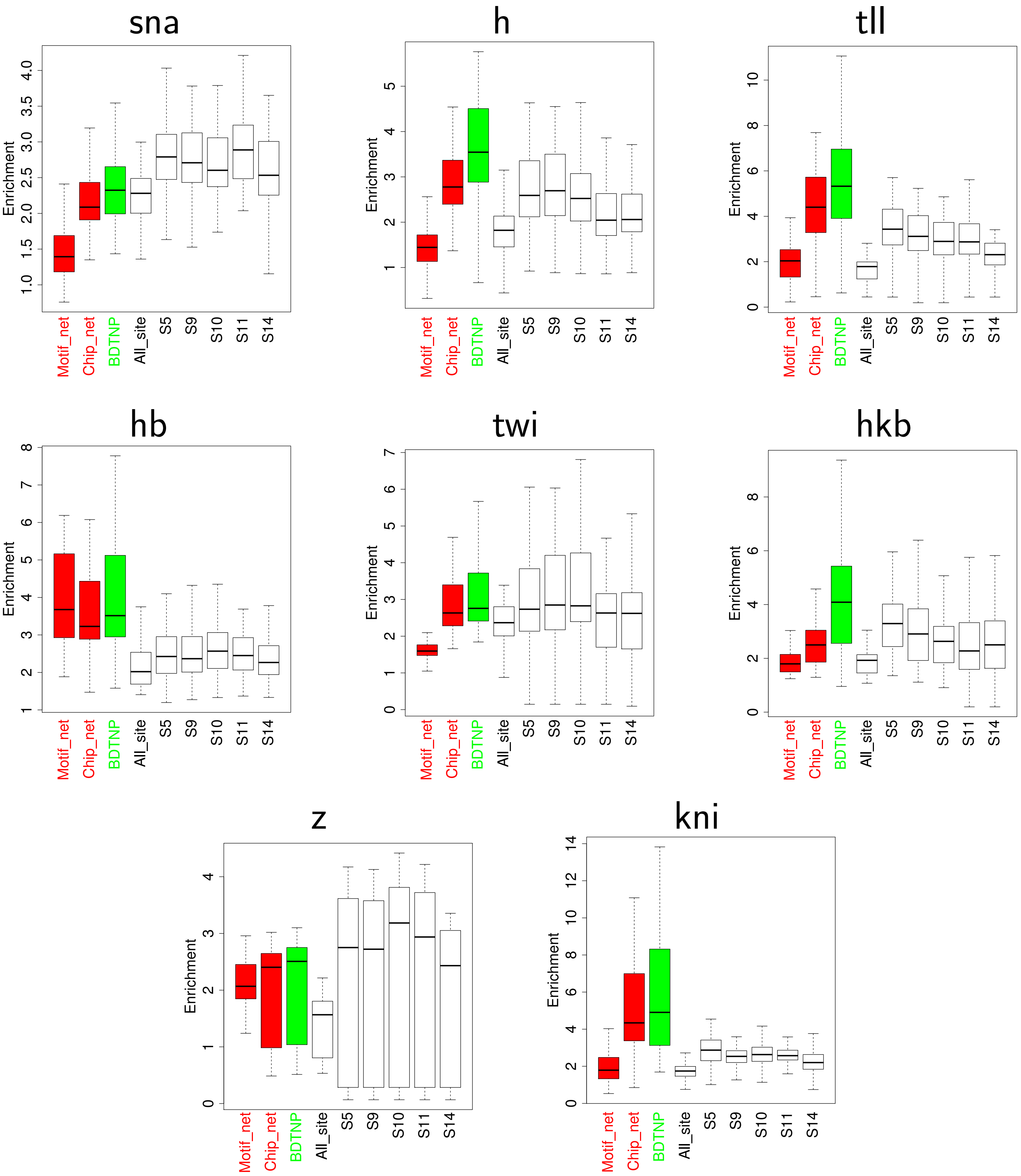

Supplementary Figure S5 - GO enrichment assessments over a range of DNaseI percentile score cutoffs

We surveyed the use of 12 different DNaseI score cutoffs, from the top 2.5% of DNaseI scores to 100% of the DNaseI scores. For each DNaseI score cutoff, we constructed the regulatory network and assessed the quality of the regulatory network by the GO enrichment measure used in Figures 2B and 2C. A conservation percentile score cutoff of 0.8 is used for this analysis. (A-E) This analysis is performed for the 17 common TFs profiled in all datasets, as in Figures 2B. (F-J) This analysis is performed for all TFs profiled in each dataset, as in Figure 2C.

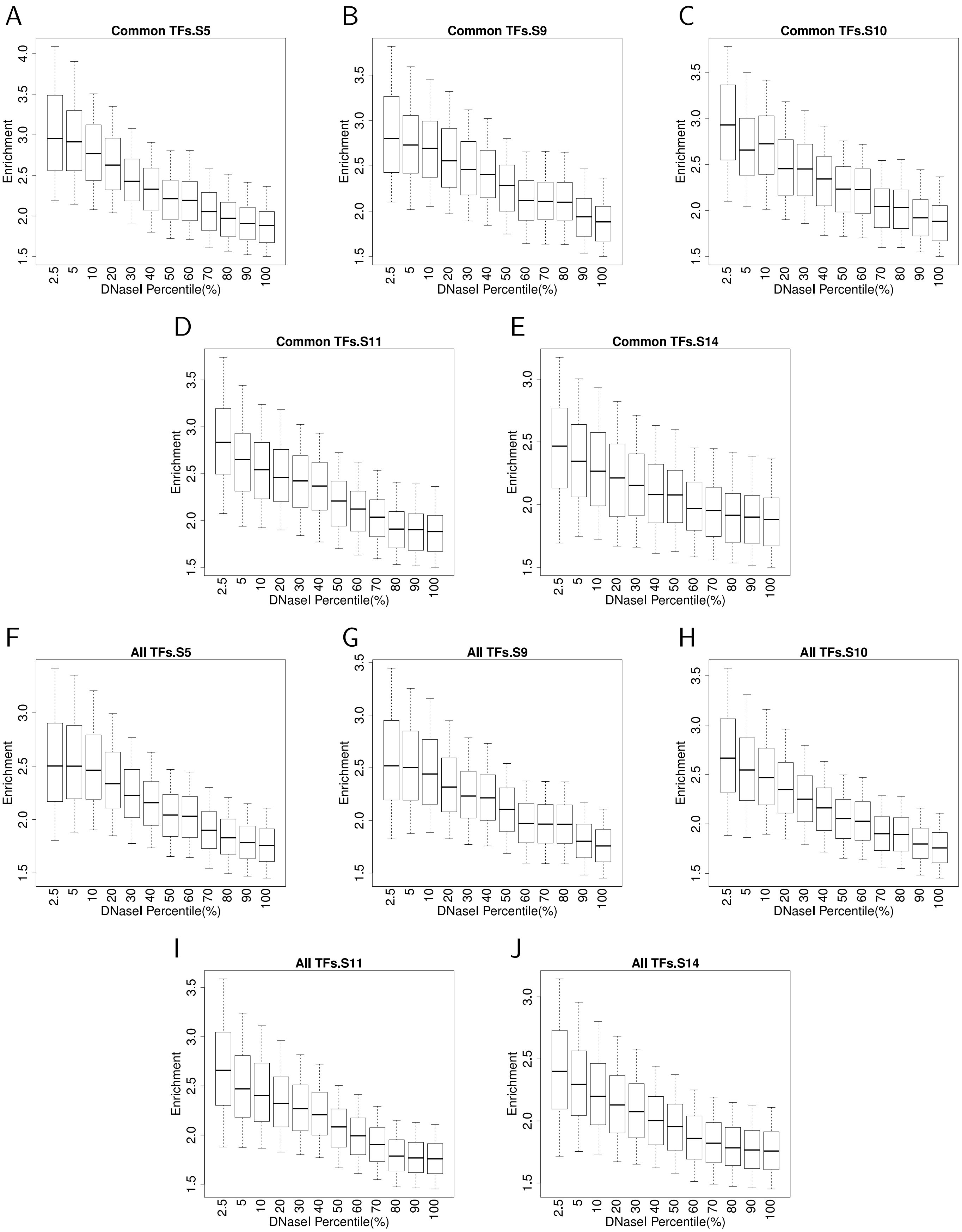
A
Common TFs.S5
B
Common TFs.S9
C
Common TFs.S10
D
Common TFs.S11
E
Common TFs.S14
F
All TFs.S5
G
All TFs.S9
H
All TFs.S10
I
All TFs.S11
J
All TFs.S14
Enrichment
DNaseI Percentile(%)
2.5 5 10 20 30 40 50 60 70 80 90 100

Supplementary Figure S6 - GO enrichment assessments for conservation percentile score cutoffs

We set up 10 different conservation percentile score thresholds, from 0.9 (most conserved) to 0.0 (least conserved). For each cutoff, we constructed the regulatory network and assessed its quality by the GO enrichment measure used in Figures 2B and 2C. A DNaseI percentile score cutoff of 5% is used for this analysis. (A-E) This analysis is performed for the 17 TFs commonly profiled in all datasets, as in Figure 2B. (F-J) This analysis is performed for all TFs profiled in each dataset, as in Figure 2C.

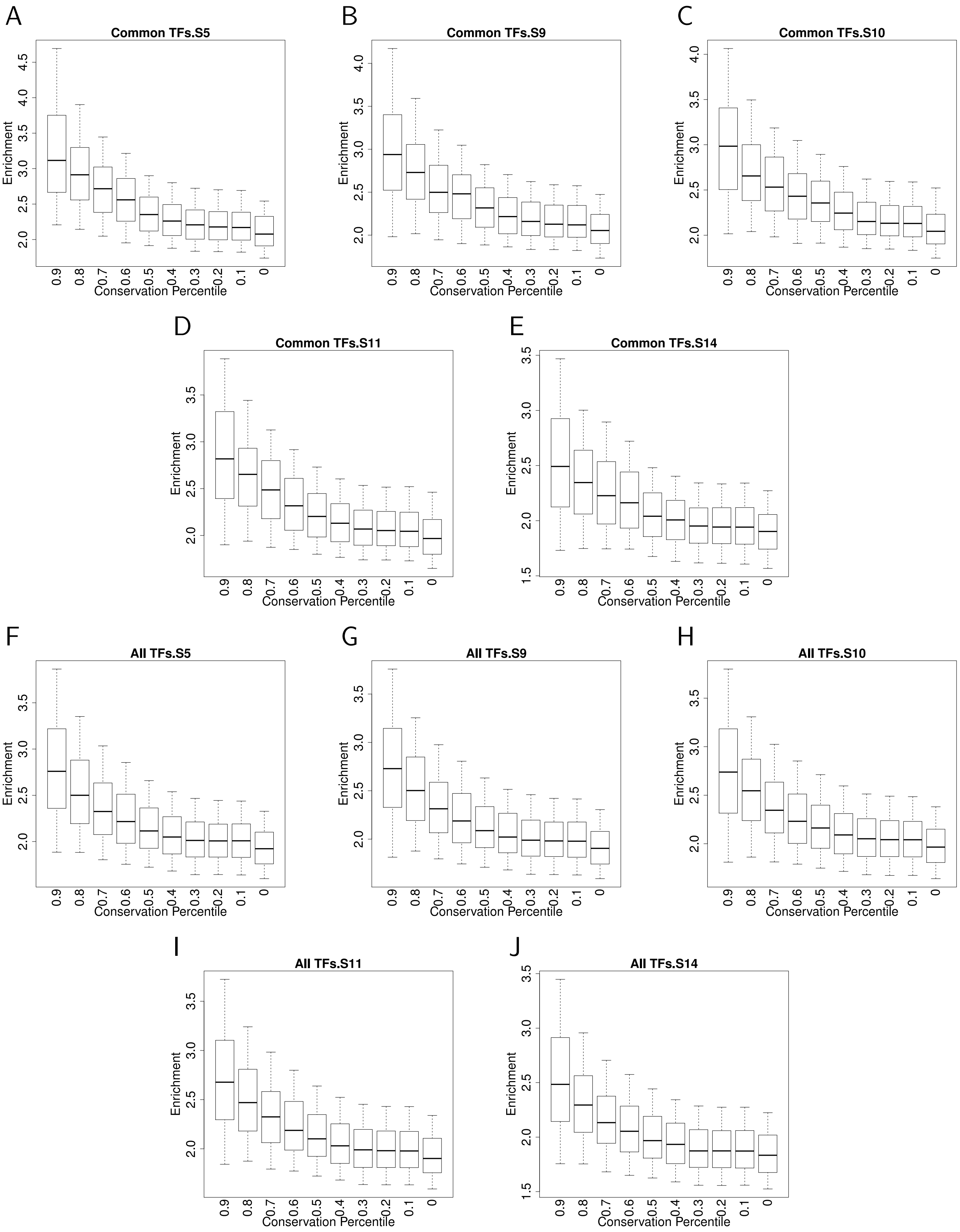

A
Common TFs.S5
B
Common TFs.S9
C
Common TFs.S10
D
Common TFs.S11
E
Common TFs.S14
F
All TFs.S5
G
All TFs.S9
H
All TFs.S10
I
All TFs.S11
J
All TFs.S14
Enrichment
Conservation Percentile
0.9 0.8 0.7 0.6 0.5 0.4 0.3 0.2 0.1 0

Supplementary Figure S7 - The CCAT regulatory network has high overlap with the Redfly dataset

505 Redfly regulatory interactions were taken as a gold standard. For each regulatory network, the interaction overlap between the it and the Redfly network was computed and compared with the overlap obtained with randomized versions of the Redfly network generated via edge swapping [1]. The enrichment ratio was defined as the real overlap count divided by the average overlap count, over 1,000 randomizations. Motif_net and ChIP_net are two networks determined by modEncode [2]. BDTNP is derived from the BDTNP ChIP experiments [3]. CCAT represents the networks predicted by our computational pipeline, which are constructed from conserved binding sites (conservation percentile score ≥0.8) over the whole genome or binding sites within DNaseI accessible regions for each developmental stage [4].

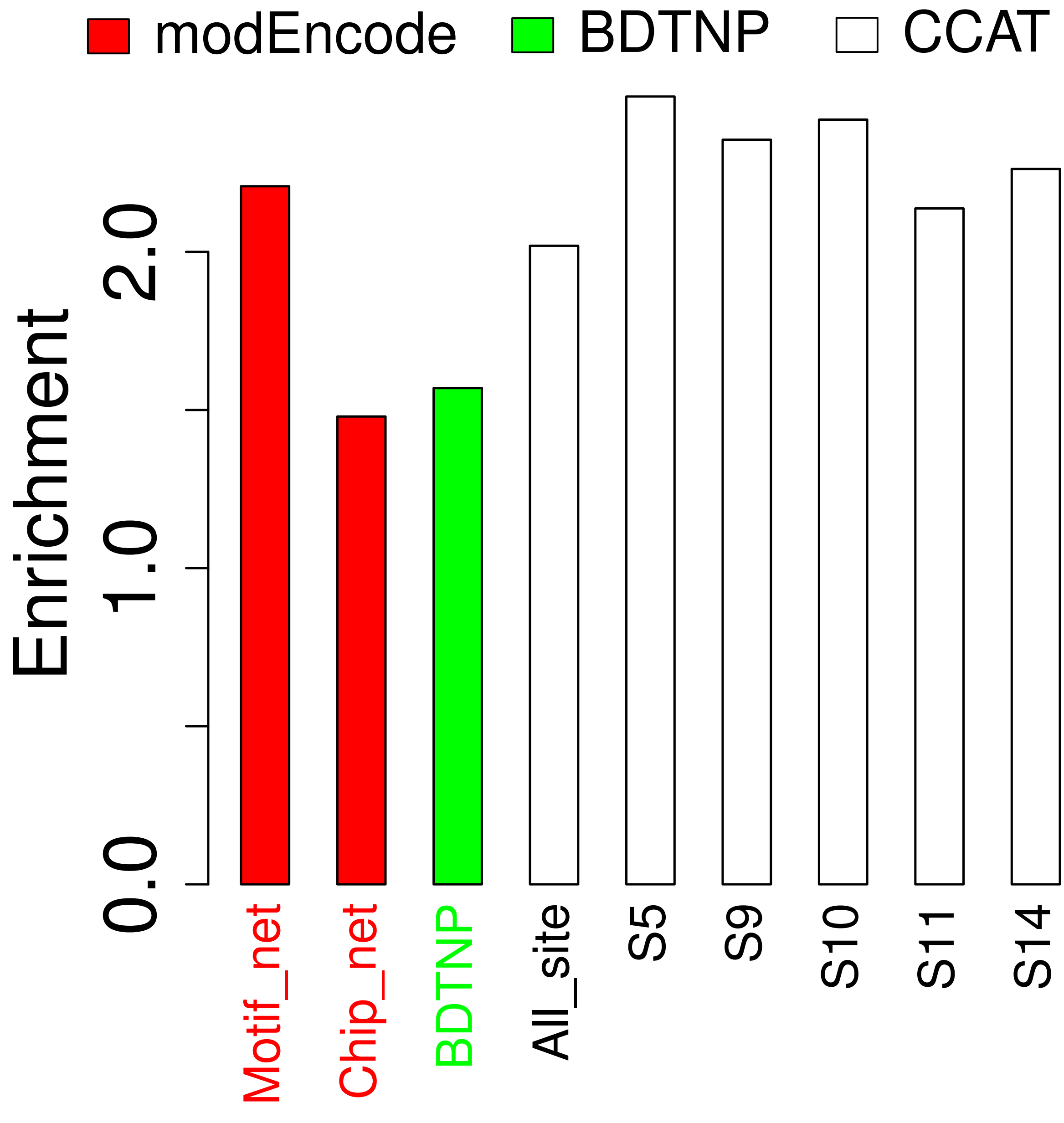

Supplementary Figure S8 - Combinatorial regulatory motif pairs

For the predicted pairs of preferentially co-localizing regulatory motifs, we plotted them as interaction graphs. (A) Stage S9. (B) Stage S10. (C) Stage S11. (D) Stage S14.

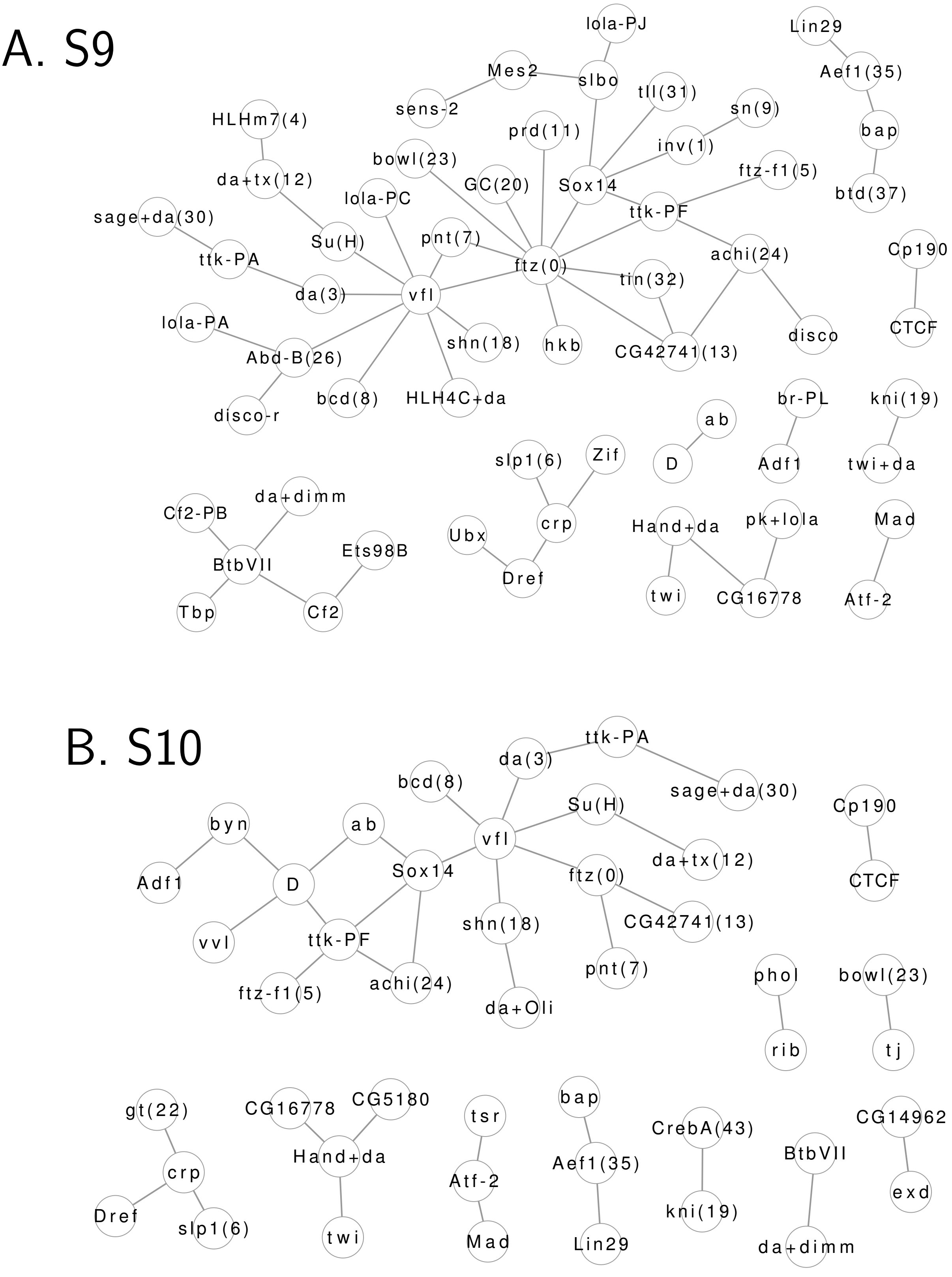

Supplementary Figure S8. continued

C. S11

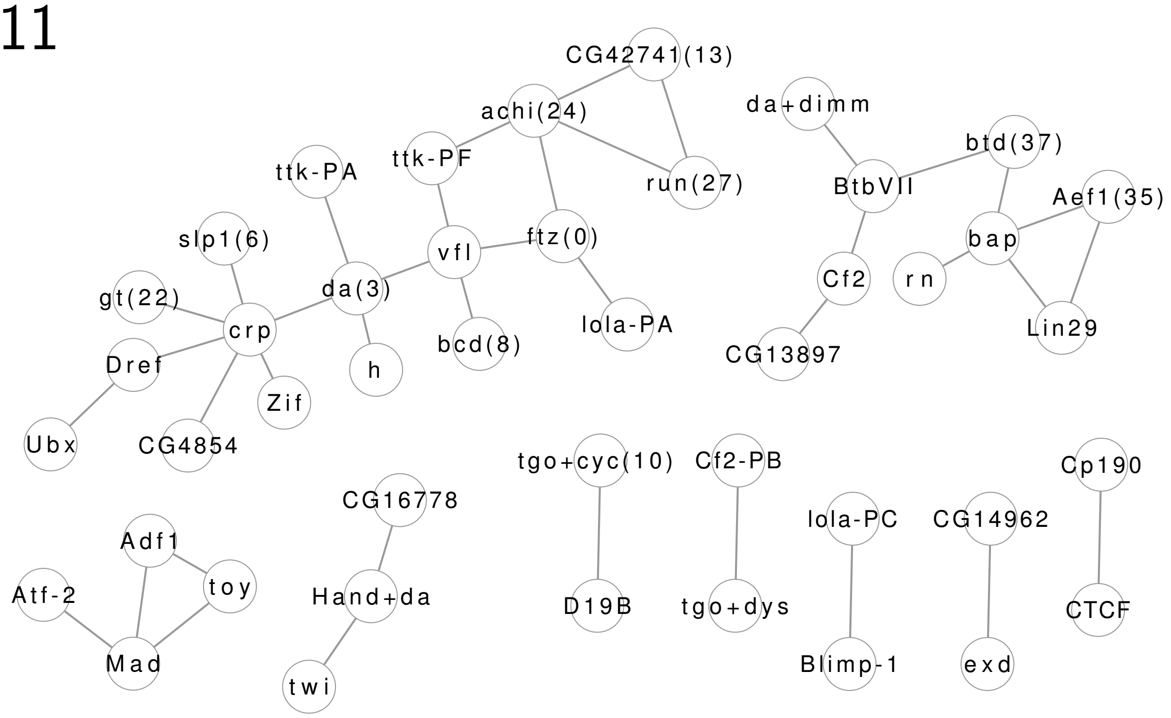

D. S14

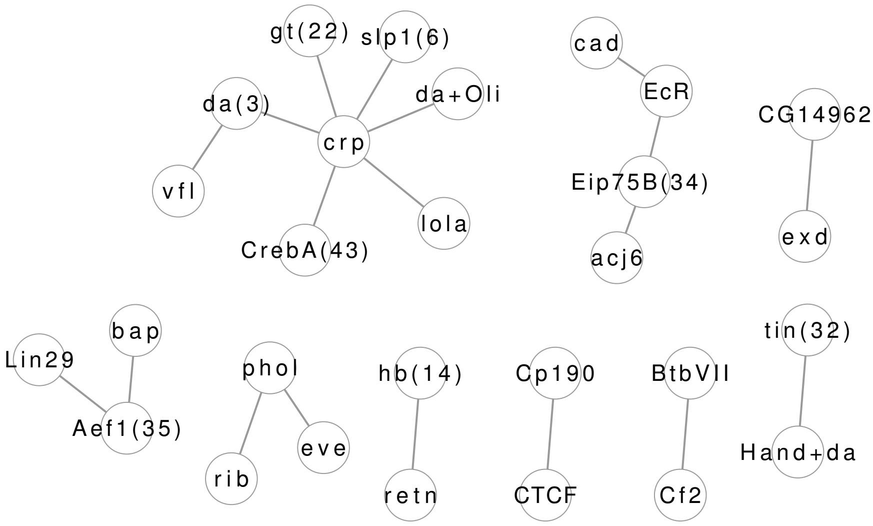

Supplementary Figure S9 - TF binding sites of combinatorial pairs are more conserved

(A) For each predicted co-localizing pair consisting of TFs A and B, the conservation percentile scores were compared among three categories of neighboring binding site predictions: (1) those with motif sites of TF A and TF B within 100 nts (TF A & TF B); (2) those with motif sites of TF A and another motif site which is not a site of TF B within 100 nts (TF A & not TF B); and (3) those with motif sites of TF B and another motif site which is not a site TF A within 100 nts (TF B & not TF A). The percent of site pairs with conservation percentile scores greater than or equal to 0.8 for both involved binding sites was calculated for all three categories. (B) For each motif pair, the measure "Difference" is defined as the percent of conserved site pairs (conservation percentile score$\geq$0.8) of category "TF A & TF B" - maximum(the percentage of category "TF A & not TF B", the percentage of category "TF B & not TF A"). For each stage, boxplots were used to visualize difference measures for all predicted co-localizing pairs and other pairs not predicted to be preferentially co-localized. The bottom and top of the box are the 25th and 75th percentiles (i.e., they give the inter-quartile range). Whiskers on the top and bottom represent the maximum and minimum data points within the range represented by 1.5 times the inter-quartile range. The Wilcoxon rank sum test was used to compare between two groups. P-values were Bonferroni corrected for the five stages. One asterisk represents a P-value $\leq$ 0.05, two asterisks represent a P-value $\leq$ 0.01 and three asterisks represent a P-value $\leq$ 0.001. (C) The regulatory motif pairs predicted in each stage were randomized by network edge swapping [1]. The median difference was plotted for real pairs and randomized pairs. The average and standard deviation values and empirical P-values were calculated from 10,000 randomizations. P-values were Bonferroni corrected for each of the five stages and visualized by asterisks as in (B).

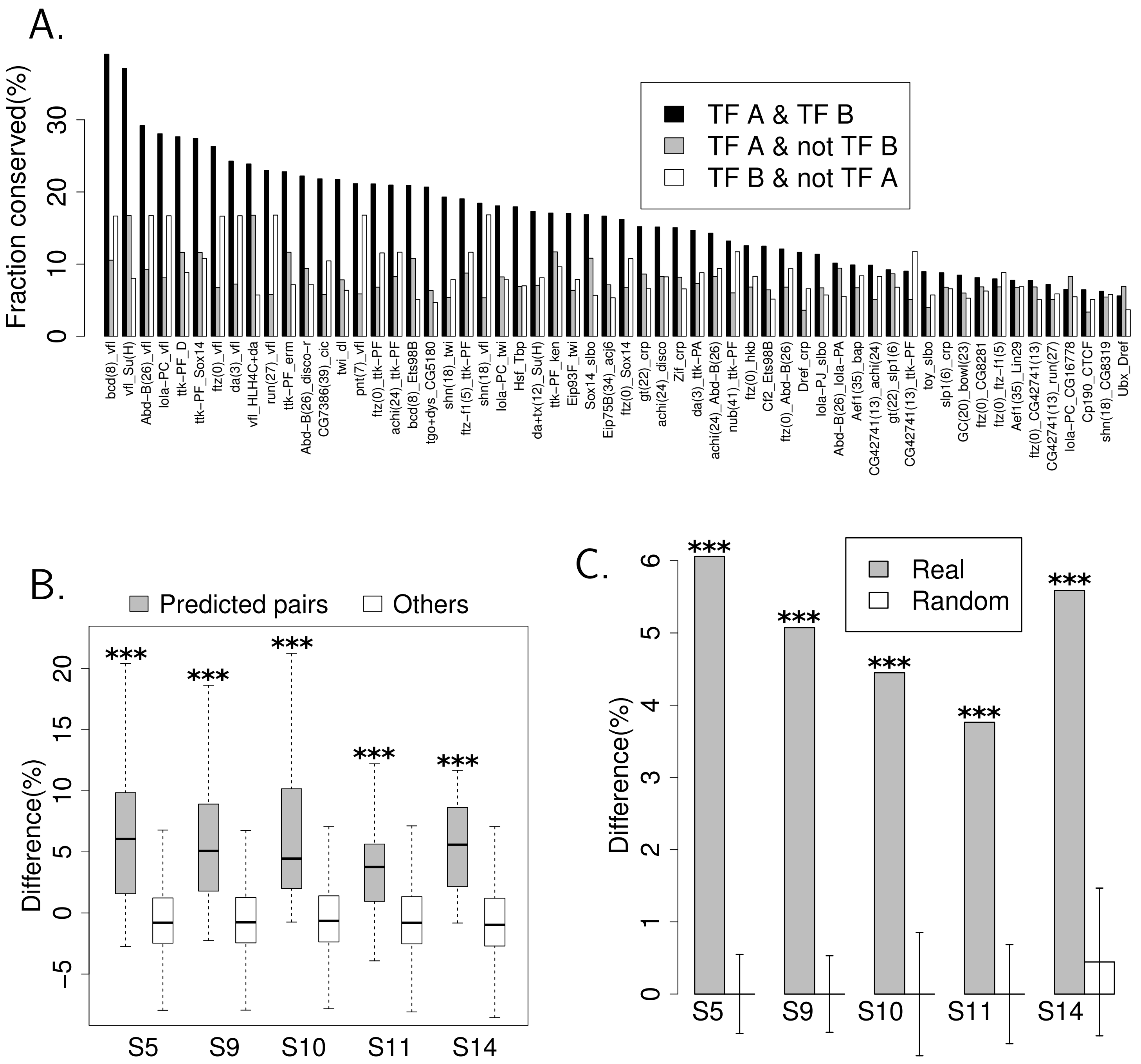
A.
Fraction conserved(%)
0
10
20
30
TF A & TF B
TF A & not TF B
TF B & not TF A
bcd(8)_vfl
vfl_Su(H)
Abd–B(26)_vfl
lola–PC_vfl
ttk–PF_D
ttk–PF_Sox14
ftz(0)_vfl
da(3)_vfl
vfl_HLH4C+da
run(27)_vfl
ttk–PF_erm
Abd–B(26)_disco–r
CG7386(39)_cic
twi_dl
pnt(7)_vfl
ftz(0)_ttk–PF
achi(24)_ttk–PF
bcd(8)_Ets98B
tgo+dys_CG5180
shn(18)_twi
ftz–f1(5)_ttk–PF
shn(18)_vfl
lola–PC_twi
Hsf_Tbp
da+tx(12)_Su(H)
ttk–PF_ken
Eip93F_twi
Sox14_slbo
Eip75B(34)_acj6
ftz(0)_Sox14
gt(22)_crp
achi(24)_disco
Zif_crp
da(3)_ttk–PA
achi(24)_Abd–B(26)
nub(41)_ttk–PF
ftz(0)_hkb
Cf2_Ets98B
ftz(0)_Abd–B(26)
Dref_crp
lola–PJ_slbo
Abd–B(26)_lola–PA
Aef1(35)_bap
CG42741(13)_achi(24)
gt(22)_slp1(6)
CG42741(13)_ttk–PF
toy_slbo
slp1(6)_crp
GC(20)_bowl(23)
ftz(0)_CG8281
ftz(0)_ftz–f1(5)
Aef1(35)_Lin29
ftz(0)_CG42741(13)
CG42741(13)_run(27)
lola–PC_CG16778
Cp190_CTCF
shn(18)_CG8319
Ubx_Dref
B.
Predicted pairs
Others
Difference(%)
−5
0
5
10
15
20
***
S5
S9
S10
S11
S14
C.
Real
Random
Difference(%)
0
1
2
3
4
5
6
***
S5
S9
S10
S11
S14

Supplementary Figure S10 - Repressor TFs may not be as enriched in accessible regions as measured by DNaseI experiments

(A) We utilized the 53 ChIP experimental datasets that we collected and checked the DNaseI score distributions for their ChIP bound regions. For each ChIP experiment, we computed the average DNaseI score for each ChIP bound region in a stage-specific manner. We next computed the percent of regions with average scores in the top 5% of DNaseI scores for each stage. The values for each stage were visualized using points of different colors and symbols. The average values among the five stages for each TF were used to rank the TFs, and a line connecting these average values is shown. We looked up the GO annotations for each TF and found 9 TFs exclusively annotated with "positive regulation of transcription, DNA-dependent"; these were used as the positive TF set and are shown with "plus" symbols in front of their gene names. We also found 10 TFs exclusively annotated with "negative regulation of transcription, DNA-dependent"; these were used as the negative TF set and are shown with "minus" symbols in front of their gene names. (B) We plotted the average fractions of ChIP bound region within DNaseI accessible region for both TF sets, and found them to be significantly different (P-value = 0.008, Wilcoxon rank sum test).

A.

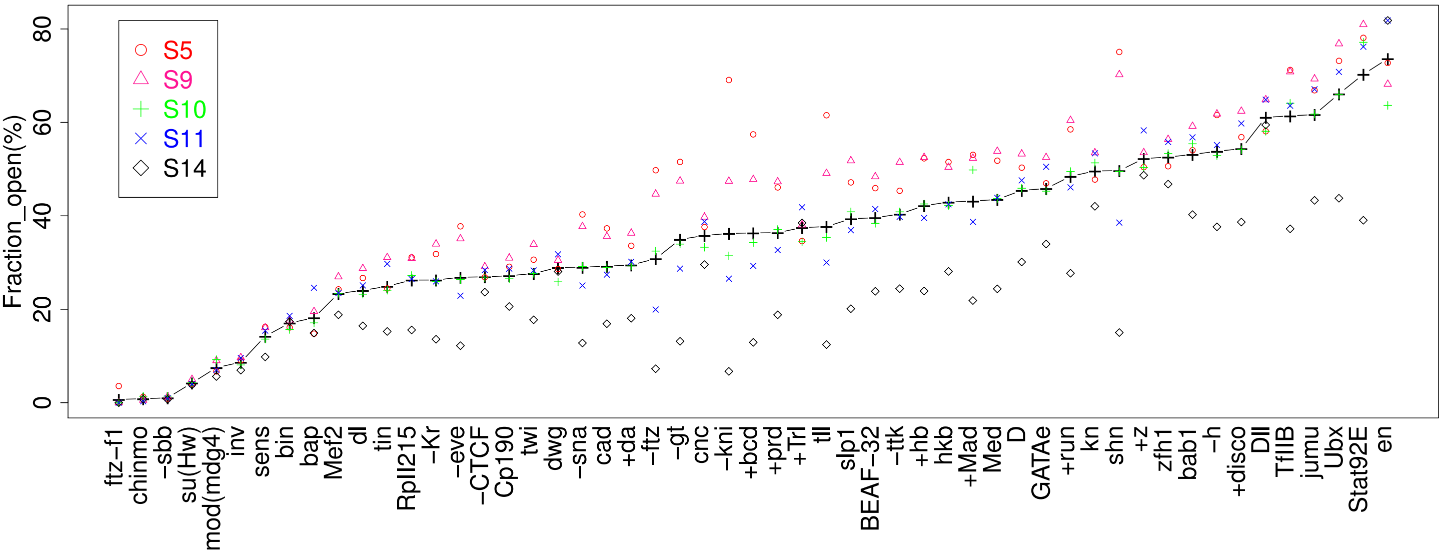

B.

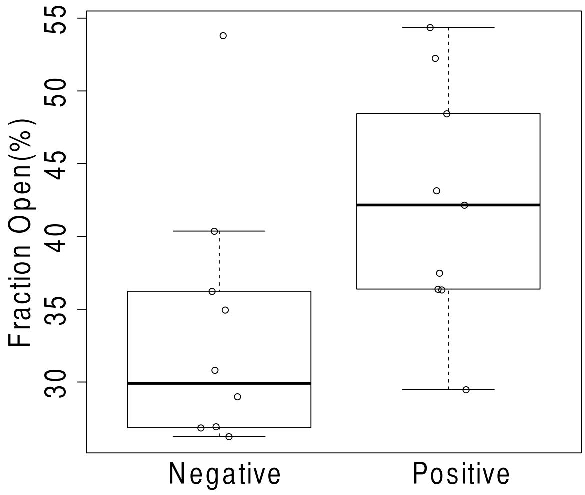

Supplementary Table S1 - Number of predicted binding sites under different thresholds

Different thresholds of DNaseI score percentile and conservation percentile are used to predict binding sites. The number of predicted binding sites using each threshold is shown for each developmental stage. (A) DNaseI percentile thresholds are varied, while a 0.8 conservation percentile score is used. (B) Conservation percentile thresholds are varied, while a DNaseI percentile threshold of 5% is used.

A.

DNaseI Percentile (%)	S5	S9	S10	S11	S14
2.5	37079	39238	34972	41060	42085
5	64275	70085	60679	74118	74735
10	99800	113975	95543	115181	135086
20	153176	173619	165186	184369	193471
30	250687	253082	263711	280016	281924
40	346858	302507	357739	343162	440146
50	507333	440982	508110	520317	569179
60	522772	663311	527631	654650	814093
70	769562	680139	754979	809425	948451
80	926156	685419	768310	1059369	1049737
90	1077321	1027212	1048800	1142457	1149551

B.

Conservation Percentile	S5	S9	S10	S11	S14
0.9	34368	37407	32500	39938	40332
0.8	64275	70085	60679	74118	74735
0.7	95545	104124	90287	109235	109411
0.6	134762	146507	127445	152658	151244
0.5	178092	193238	168521	199956	196479
0.4	215773	233893	204790	240713	234674
0.3	242769	262856	230388	269651	261726
0.2	250514	271138	237853	278192	270233
0.1	251325	272035	238680	279111	271071
0.0	322265	347832	308794	354144	342748

Supplementary Table S2 - Coherence with ChIP dataset under different DNaseI percentile thresholds

Binding sites were predicted using different DNaseI percentile thresholds while using a conservation percentile score of 0.8. For each profiled TF that has a corresponding ChIP dataset, we took the union of all predicted binding sites across the five stages, and calculated the percent of these sites that are located within an experimentally identified bound region.

DNaseI Percentile (%)	2.5	5	10	20	30	40	50	60	70	80	90	100
Trl	88.28	83.24	77.26	71.20	65.48	60.75	56.52	53.20	50.97	49.88	48.88	47.88
dl	85.11	81.10	71.66	60.90	49.75	41.60	35.14	30.46	27.73	26.26	25.45	25.11
twi	81.09	77.11	67.46	57.76	48.74	41.33	35.63	31.99	29.88	29.02	28.47	28.09
D	78.85	67.13	53.98	42.58	32.27	25.75	20.92	17.42	15.76	14.88	14.29	14.15
da	71.87	65.47	56.85	47.49	38.27	31.28	26.71	23.42	21.82	20.93	20.47	20.35
Kr	63.08	64.04	56.60	49.42	41.67	36.30	32.62	29.35	27.41	26.24	25.75	25.54
cad	65.43	57.55	48.13	39.54	30.52	25.58	21.43	18.11	16.25	15.18	14.29	14.13
su(Hw)	51.75	56.41	59.35	59.10	59.15	58.06	56.96	55.70	54.94	54.60	54.29	54.26
Cp190	59.80	51.49	42.04	33.76	27.55	23.63	21.23	19.60	18.65	18.39	17.96	17.93
Med	61.22	49.66	36.17	26.75	20.04	16.04	13.02	10.96	9.96	9.47	9.21	9.13
h	55.86	46.86	36.18	27.41	21.99	17.39	14.54	12.68	11.74	11.22	10.95	10.86
prd	49.45	41.30	33.62	26.74	20.40	15.98	13.61	11.74	10.76	10.29	9.98	9.63
z	49.64	39.70	29.32	20.52	14.61	11.22	8.83	7.42	6.69	6.30	6.06	6.00
bcd	47.14	38.83	30.22	21.10	15.37	11.39	8.93	7.38	6.56	6.18	5.95	5.87
sens	48.10	38.37	29.57	23.32	18.70	14.27	11.80	9.83	8.73	8.23	7.87	7.79
CTCF	36.86	36.79	31.49	27.61	23.64	21.33	20.20	19.11	18.30	17.96	17.78	17.82
hb	38.69	32.92	26.08	20.00	15.25	12.10	9.60	7.75	6.65	6.05	5.50	5.30
sna	41.10	32.31	25.18	19.68	15.39	12.29	10.43	9.16	8.52	8.16	7.97	7.93
run	38.87	30.08	20.26	13.77	9.05	6.55	5.02	3.97	3.51	3.28	3.15	3.12
hkb	37.58	28.95	19.44	13.34	9.58	7.07	5.71	4.77	4.26	4.06	3.91	3.85
Mef2	24.64	23.02	19.31	14.75	11.08	9.24	8.42	7.26	6.69	6.61	6.36	6.29
gt	24.20	20.25	15.94	11.88	9.08	7.22	5.82	4.95	4.56	4.32	4.20	4.17
slp1	21.93	17.27	13.06	9.65	7.21	5.68	4.59	3.90	3.51	3.32	3.19	3.15
tin	20.95	16.17	11.23	7.77	5.65	4.54	3.71	3.31	3.09	2.98	2.90	2.88
shn	20.16	14.79	9.42	6.53	4.43	3.30	2.61	2.18	1.98	1.85	1.79	1.76
tll	18.44	14.17	10.13	7.17	5.08	3.68	2.82	2.25	2.01	1.86	1.79	1.77
disco	20.62	13.51	7.94	5.69	4.02	2.88	2.30	1.89	1.71	1.61	1.56	1.55
Ubx	14.61	10.61	7.29	4.74	3.15	2.37	1.75	1.36	1.15	1.04	0.94	0.90
Stat92E	11.48	7.93	5.20	3.67	2.65	1.98	1.53	1.22	1.10	1.05	1.00	0.98
bin	7.94	7.39	6.82	5.78	4.68	4.03	3.43	3.04	2.80	2.67	2.58	2.56
ftz	10.72	6.68	4.08	2.48	1.64	1.17	0.86	0.68	0.58	0.54	0.51	0.51
eve	7.81	6.50	4.10	2.99	2.20	1.65	1.39	1.13	1.02	0.96	0.93	0.92
bab1	6.14	6.20	4.90	3.91	2.64	2.22	1.75	1.44	1.25	1.15	1.06	1.04
kni	6.32	5.13	3.93	3.16	2.23	1.71	1.37	1.14	1.04	0.99	0.96	0.95
Mad	6.48	4.50	3.34	2.43	1.83	1.50	1.28	1.10	1.01	0.96	0.94	0.92
inv	2.96	3.74	3.88	3.94	3.53	3.35	3.19	3.11	3.12	3.07	3.04	3.02
bap	2.38	2.10	1.64	1.35	1.19	1.04	0.92	0.88	0.84	0.82	0.81	0.80
GATAe	2.01	1.06	0.81	0.64	0.51	0.44	0.37	0.33	0.31	0.30	0.29	0.29
ttk	0.21	0.12	0.15	0.12	0.09	0.08	0.07	0.06	0.06	0.06	0.06	0.05

Supplementary Table S3 - Coherence with ChIP dataset under different conservation percentile thresholds

Binding sites were predicted using different conservation percentile score thresholds and a DNaseI precentile score cutoff of 5%. For each profiled TF with a corresponding ChIP dataset, we took the union of all predicted binding sites across the five stages, and calculated the percent of these sites that are located within an experimentally identified bound region.

Conservation percentile	0.0	0.1	0.2	0.3	0.4	0.5	0.6	0.7	0.8	0.9
Trl	74.22	76.83	76.83	77.32	78.55	79.80	82.29	82.04	83.24	85.32
dl	77.83	80.17	80.17	80.16	80.42	80.21	80.78	81.48	81.10	80.79
twi	69.69	72.82	72.83	73.47	74.67	75.88	76.80	77.61	77.11	79.72
D	65.55	68.02	68.02	68.02	68.00	67.06	66.48	66.48	67.13	65.79
da	55.99	59.59	59.58	61.22	62.27	63.92	64.94	65.63	65.47	67.38
Kr	50.44	52.59	52.59	52.59	52.74	54.28	57.22	62.50	64.04	67.12
cad	41.12	48.00	48.00	48.00	48.00	50.08	50.42	57.23	57.55	62.57
su(Hw)	25.28	30.17	30.19	30.19	34.30	35.60	41.46	50.14	56.41	64.96
Cp190	51.90	56.84	56.84	56.82	56.82	57.85	60.26	60.41	51.49	51.58
Med	52.52	53.96	53.96	54.38	54.55	53.95	53.68	50.22	49.66	48.65
h	37.20	39.52	39.53	40.29	41.53	43.45	43.50	45.88	46.86	46.98
prd	26.51	30.02	30.17	30.25	31.84	34.83	37.32	38.54	41.30	45.20
z	25.83	28.76	28.76	28.76	31.93	32.08	34.25	38.00	39.70	38.52
bcd	21.26	26.22	26.48	26.43	27.82	29.68	31.47	36.50	38.83	42.81
sens	33.51	37.23	37.23	37.23	37.25	38.63	39.50	37.97	38.37	38.89
CTCF	25.27	29.25	29.26	29.27	32.60	34.61	37.15	35.34	36.79	42.91
hb	26.64	30.07	30.21	30.43	30.59	31.07	31.59	32.69	32.92	32.47
sna	24.89	26.76	26.75	27.96	28.98	30.07	30.28	31.12	32.31	34.20
run	13.68	15.75	15.77	15.92	18.18	20.36	25.56	25.47	30.08	33.12
hkb	18.90	21.19	21.19	21.77	23.15	24.29	28.16	28.45	28.95	30.79
Mef2	12.54	14.29	14.29	14.29	14.29	14.58	16.06	20.41	23.02	19.75
gt	14.70	17.18	17.21	17.16	17.43	18.16	18.82	19.98	20.25	20.82
slp1	13.29	14.58	14.61	14.50	14.87	15.06	15.62	16.81	17.27	18.36
tin	10.30	11.57	11.62	12.61	13.31	15.66	16.20	16.33	16.17	19.52
shn	6.62	7.83	7.93	7.95	8.98	9.41	10.20	10.54	14.79	16.96
tll	7.60	9.04	9.08	9.15	9.54	10.50	11.43	13.74	14.17	13.35
disco	10.90	11.33	11.33	11.33	11.29	11.59	12.26	12.29	13.51	17.06
Ubx	16.97	15.07	15.07	15.07	15.10	15.13	15.13	13.04	10.61	5.14
Stat92E	3.00	3.73	3.73	3.69	3.70	4.34	4.67	5.15	7.93	8.80
bin	4.91	5.44	5.44	5.44	5.68	5.98	6.58	7.11	7.39	7.36
ftz	3.17	3.86	3.90	3.99	4.31	4.74	5.43	6.12	6.68	6.56
eve	2.91	3.11	3.11	3.12	3.45	3.49	3.68	6.62	6.50	8.85
bab1	11.50	9.91	9.91	9.92	9.93	9.23	7.78	6.48	6.20	3.88
kni	2.91	3.24	3.26	3.28	3.53	3.58	4.21	4.83	5.13	6.15
Mad	2.32	2.62	2.63	2.83	2.89	3.30	3.41	3.48	4.50	4.00
inv	3.85	4.06	4.09	4.11	4.16	3.93	4.26	4.02	3.74	3.38
bap	2.00	2.06	2.07	2.06	2.07	2.08	2.14	2.17	2.10	2.36
GATAe	2.44	2.48	2.49	2.47	2.14	1.92	1.34	1.28	1.06	0.91
ttk	0.59	0.52	0.52	0.52	0.52	0.29	0.30	0.16	0.12	

References

[1] Milo R, Kashtan N, Itzkovitz S, Newman MEJ, Alon U: **On the uniform generation of random graphs with prescribed degree sequences**. *arXiv* 2004.

[2] Roy S, Ernst J, Kharchenko PV, Kheradpour P, et al.: **Identification of functional elements and regulatory circuits by Drosophila modENCODE**. *Science* 2010, **330**(6012):1787–1797.

[3] MacArthur S, Li XY, Li J, Brown JB, Chu HC, Zeng L, Grondona BP, Hechmer A, Simirenko L, KerÃďnen SV, Knowles DW, Stapleton M, Bickel P, Biggin MD, Eisen MB: **Developmental roles of 21 Drosophila transcription factors are determined by quantitative differences in binding to an overlapping set of thousands of genomic regions**. *Genome Biol* 2009, **10**(7):R80.

[4] Thomas S, Li XY, Sabo PJ, Sandstrom R: **Dynamic reprogramming of chromatin accessibility during Drosophila embryo development**. *Genome Biol* 2011, **12**(5):R43.
